# The molecular evolution of cancer associated genes in mammals

**DOI:** 10.1038/s41598-024-62425-0

**Published:** 2024-05-22

**Authors:** Nick MacDonald, Nynke Raven, Wendy Diep, Samantha Evans, Senuri Pannipitiya, Georgina Bramwell, Caitlin Vanbeek, Frédéric Thomas, Tracey Russell, Antoine M. Dujon, Marina Telonis-Scott, Beata Ujvari

**Affiliations:** 1 School of Life and Environmental Sciences, Deakin University, Geelong, Waurn Ponds, Geelong, VIC 3216 Australia; 2 CREEC, UMR IRD 224-CNRS 5290, Université de Montpellier, Montpellier, France; 3 MIVEGEC, IRD, CNRS, Université Montpellier, Montpellier, France; 4 Faculty of Science, School of Life and Environmental Sciences, Sydney, NSW Australia; 5 School of Life and Environmental Sciences, Deakin University, Burwood, Burwood, VIC 3125 Australia

**Keywords:** Cancer susceptibility, Comparative genomics, Mammals, Bioinformatics, Evolution, HyPhy, Phylogenetics, Cancer genetics

## Abstract

Cancer is a disease that many multicellular organisms have faced for millions of years, and species have evolved various tumour suppression mechanisms to control oncogenesis. Although cancer occurs across the tree of life, cancer related mortality risks vary across mammalian orders, with Carnivorans particularly affected. Evolutionary theory predicts different selection pressures on genes associated with cancer progression and suppression, including oncogenes, tumour suppressor genes and immune genes. Therefore, we investigated the evolutionary history of cancer associated gene sequences across 384 mammalian taxa, to detect signatures of selection across categories of oncogenes (GRB2, FGL2 and CDC42), tumour suppressors (LITAF, Casp8 and BRCA2) and immune genes (IL2, CD274 and B2M). This approach allowed us to conduct a fine scale analysis of gene wide and site-specific signatures of selection across mammalian lineages under the lens of cancer susceptibility. Phylogenetic analyses revealed that for most species the evolution of cancer associated genes follows the species’ evolution. The gene wide selection analyses revealed oncogenes being the most conserved, tumour suppressor and immune genes having similar amounts of episodic diversifying selection. Despite BRCA2’s status as a key caretaker gene, episodic diversifying selection was detected across mammals. The site-specific selection analyses revealed that the two apoptosis associated domains of the Casp8 gene of bats (Chiroptera) are under opposing forces of selection (positive and negative respectively), highlighting the importance of site-specific selection analyses to understand the evolution of highly complex gene families. Our results highlighted the need to critically assess different types of selection pressure on cancer associated genes when investigating evolutionary adaptations to cancer across the tree of life. This study provides an extensive assessment of cancer associated genes in mammals with highly representative, and substantially large sample size for a comparative genomic analysis in the field and identifies various avenues for future research into the mechanisms of cancer resistance and susceptibility in mammals.

## Introduction

Although many multicellular organisms face the threat of cancer^1^, differences in environment, life history traits and behaviour can influence an organism’s propensity for cancer risk^2^. In theory, those that are particularly susceptible to neoplasm should have evolved better resistance to cancer^3^. For example, it would be expected that long-lived, larger bodied species, would have a higher risk of cancer due to increased cell division^4^. However, large animals like the bowhead whale (*Balaena mysticetus*), and relatively long-lived animals like the naked mole rat (*Heterocephalus glaber*) exhibit fewer incidences of cancer^5,6^. This disparity has been coined as Peto’s paradox^7,8^. Several studies^8–11^ have addressed Peto’s paradox, finding that increases in body size and life span are accompanied by adaptations to reduce cancer risk. In captive animals, cancer mortality risk is largely independent of both body mass and adult life expectancy across species^12^, while the main risk factors are environmental, particularly diet. Carnivorous mammals have been found to have the highest cancer related mortality risk compared to the least cancer-prone mammalian order, Artiodactyla (even-toed ungulates)^12^.

Long-lived and large species have evolved a plethora of anticancer defences and strategies to supress tumour development^13–15^. Many of the adaptations include variation in gene expression, genomic composition and chromosome maintenance. This includes copy number changes in tumour suppressor genes (TP53) of elephants^16,17^ and duplication of DNA repair genes in whales^5^. However, the functional importance of TP53 duplications in elephants is under debate^18^, as tumour suppressor and oncogene copy number have been shown to be positively correlated with lifespan (e.g. highest cancer gene copies in naked mole rats) but not body size across 63 mammalian genomes^19^. The role of gene expression in cancer has been explored with miRNA, and transcriptomic profiles associated with longevity have been identified in bat species^20^. Bats also having shown distinct evolutionary patterns in cancer associated genes^21^. Additionally, the regulation of telomere maintenance has been recognised in the evolution of larger species for cancer prevention^22–24^.

Comparative genomics has highlighted both negative and positive selection acting on cancer associated genes across mammalian evolution driving these adaptations^25–29^, with the strength and direction depending on the species and the category and function of the gene. In the context of cancer, there are three, non-exclusive categories of genes: proto-oncogenes (hereforth referred to as oncogenes), tumour suppressor genes (TSG) and immune genes. The trends in selection pressure on the genes in the three categories are influenced by their role in cancer progression and their normal physiological function. When a mutation occurs, proto-oncogenes can become oncogenes, where their general functions are exploited by proliferating cancer cells^30^. For most neoplasia, a mutation in only one copy of an oncogene is required to initiate cancer development^31^. This results in strong negative selection on the genes and proto(oncogenes) along with TSGs are often highly conserved^32^.

Conservation of both onco- and tumour suppressor genes is important in preventing cancer, however the selection pressure on oncogenes is stronger^27^. The mutational targets of oncogenes and TSGs differ, as mutations in oncogenes are dominant, while changes in TSGs are recessive^27^. While inactivation of only one allele of an oncogene is enough to contribute to tumorigenesis, the inactivation of both alleles are required for TSGs^31^.The requirement for mutations in two copies is known as Knudson’s two-hit hypothesis^33^. Additionally, oncogenes often arise from genes involved in essential cellular functions, making their conservation important^34^. However, cancer progression is more complex than simply a one/two-hit hypothesis, as mutations in multiple cancer associated genes are needed for neoplastic progression^35^. Additionally, mutations in genes may also depend on protein–protein interactions with other genes, adding complexity to the two-hit hypothesis^36,37^.

Finally, the immune system and associated immune genes play important roles in cancer development and progression. Both the adaptive and innate arms of the immune system include mechanisms to detect and remove cancer cells^38^. While many immune genes can be classified as such, the actions of immune cells are governed by multiple signals and the role of some immune genes in cancer development is more complex than simply promoting or suppressing neoplasia^39,40^. The immune system also has a key role in mitigating the impact of potential cancer causing pathogens, including viruses, bacteria, and parasites^41,42^. As a result, immune genes are one of the most genetically variable groups of genes in the genome^29,43^.

The recent expansion of genome sequencing projects makes it possible for a more focused view on the evolution of cancer associated genes. This study, therefore, aimed to discern the evolutionary history of cancer associated genes as well as the selection pressures operating on them across mammalian subgroups. By limiting the number of targeted genes to nine (i.e., oncogenes (GRB2, FGL2 and CDC42), tumour suppressor genes (LITAF, Casp8 and BRCA2) and immune genes (CD274, IL2 and B2M)), we were able to expand the number of species included in the analysis^1^. The genes were selected based on their role in different aspects of tumour formation and growth. For example, the products of oncogenes GRB2 and CDC42 direct the cells to grow and expand beyond their tissue of origin^44,45^. Proteins produced by the FGL2 gene can promote angiogenesis^46^ to engineer the tumours’ environment in a way that can facilitate growth and movement. Tumour suppressor genes can have direct roles in influencing tumour growth, for example CASP8’s function as a gene in the apoptosis pathway^47^, while the proteins generated from BRCA2 prevent cells from becoming cancerous, through maintaining genomic stability^48^. The expression of genes such as LITAF can be a sign of a positive cancer prognosis and survival^49^. Cytokines and signalling molecules have been used by cancer cells to regulate the immune system in their favour, for example tumours can express CD274 in order to directly supress T-cell activity^50^. Tumour immunity is however a complex process, for example the expression of IL2 can both promote T-reg activity for the benefit of tumours, but it can also promote the activity of anti-tumour T-cells^51^. Another strategy is immune evasion through the downregulation of potential tumour associated antigens, for example via the downregulation of B2M, which is involved in antigen presentation^52^.

By limiting the number of genes analysed, but substantially expanding the number of species included, the study was able to (i) create a representative sample of the available mammalian genomes and (ii) conduct a fine scale analysis of site-specific signatures of selection across mammalian lineages under the lens of cancer susceptibility.

## Materials and methods

### Glossary of terms

Monophyletic—group of organisms that share a common ancestor.

Polytomy—a node on a phylogeny where more than two lineages descend from a single ancestral lineage.

Pervasive positive selection—positive selection occurring on multiple members of an evolutionary branch.

Pervasive negative selection—negative selection occurring on multiple members of an evolutionary branch.

Episodic positive selection—selection pressure that will occur on only select members of an evolutionary branch, often due to sudden changes in the ecosystem.

### Gene selection

Based on their roles associated with cancer development and progression, nine genes were selected for in depth analyses. Three oncogenes; Growth factor receptor-bound protein 2 (GRB2), Fibrinogen like protein 2 (FGL2) and Cell Division Control protein 42 homolog (CDC42) and three tumour suppressors; Lipopolysaccharide Induced Tumour necrosis Factor (LITAF), Caspase 8 (Casp8) and Breast Cancer gene 2 (BRCA2), and three immune genes; Interleukin-2 (IL2), Cluster Differentiation 274 (CD274), and Beta-2-Microglobin (B2M) (functional descriptions are provided in Table 1).

**Table 1:** Description of the cancer associated genes analysed in the study.

Gene	Abbreviation	Category	Function
Growth factor receptor bound protein 2	GRB2	Oncogene	Involved in cell cycle progression, cell motility, morphogenesis, angiogenesis
Fibrinogen like protein 2	FGL2	Oncogene	Promotes angiogenesis Acts as an immunosuppressor
Cell Division Control protein 42 homolog	CDC42	Oncogene	Involved in cell morphology, migration and cell cycle progression
Lipopolysaccharide induced tumour necrosis factor	LITAF	Tumour suppressor gene	Implicated in the P53 signalling pathway, low expression is associated with cancer
Caspase 8	Casp8	Tumour suppressor gene	Involved in the execution of the apoptotic pathway
Breast Cancer gene 2	BRCA2	Tumour suppressor gene	Involved in DNA repair and homologous recombination
Interleukin 2	IL2 somatic	Immune gene	Promotes the proliferation of T-cells
Cluster differentiation 274	CD274 somatic	Immune gene	Inhibits T-cell activity
Beta-2-Microglobin	B2M	Immune gene	Structural component of the Class 1 Major Histocompatibility molecules

Description of genes were obtained from the Genecards website^108^.

### Study species, genome sources and tools

A total of 386 mammalian species were included in the analyses from genomes available in the NCBI^53^ and Ensembl^54^ databases (Data accessed from April 2020 through to March 2021, Ensembl release 101). See Table 2 for details, and Supplementary S2 for gene ID’s. Sequences were obtained from the orthologues available for each gene in the aforementioned databases. Sequences for species without available orthologues were retrieved by searching the whole genomes with the NCBI’s Basic Local Alignment Search Tool (BLAST)^55^. Exons were extracted and gene sequences were constructed and annotated manually. Species with poorly sequenced genomes, not yielding BLAST results or with gene sequences unavailable across more than two genes were not included in the analysis.

**Table 2:** Mammalian *taxa* and number of species per subgroups used in the study.

Taxa	Level of classification	Number of species
Mammals	Class	386
Afrotheria	Superorder	9
Artiodactyla	Order	104
Carnivora	Order	54
Chiroptera	Order	39
Marsupial	Infraclass	7
Perissodactyla	Order	8
Primate	Order	57
Rodentia	Order	86
Xenarthra	Superorder	7
Eulipotyphla	Order	4
Lagomorpha	Order	4
Pholidota	Order	3
Sundatheria	Clade	3

As an outgroup, a species of sauropsid was used. As no single sauropsid species had an available annotation for every gene, different species were used as outgroups in some of the analyses. Sauropsids were chosen as they are the closest relatives to mammals. Given all sauropsid species are equally distantly related to mammals, the use of multiple species is unlikely to impact the results and the specific sauropsid used is arbitrary^56^. The green sea turtle (*Chelonia mydas*) was used as the outgroup in the phylogenetic analysis of GRB2, FGL2, CDC42, LITAF, BRCA2, CD274 and B2M, the painted turtle (*Chrysemys picta*) for Casp8 and the chicken (*Gallus gallus*) for IL2 (Supplementary S2).

Multiple sequence alignments were conducted on the entire coding region of each gene using Bioedit V7.2^57^. The length of the human gene was used as a standard, as it is the most well studied species and the multiple sequence alignments were trimmed to match the length of the human sequence. Each sequence was manually checked for assembly and annotation errors, and introns retained during the process of automated genome annotation were removed from the sequences. As the downstream analysis required the use of coding sequences the terminal stop codons were also removed from the 3’ end of the sequence.

### Reconstruction of phylogenetic relationships

Evolutionary relationships for each gene were examined using phylogenetic trees constructed in MEGA-X^58^. Phylogenetic trees were modelled using Kimura 2-parameter model^59^, and maximum likelihood analyses were run with 500 bootstrap replications and a bootstrap cut off value of 70 on the final tree. Nearest neighbour joining tree inference was used. The phylogenetic trees were visualised using iTol V5^60^.

To identify significant deviations in the evolution of genes compared to the evolution of species, a control phylogenetic tree was constructed based on current mammalian phylogenetic relationships. A pruned control tree was obtained from the phylogenetic database Vertilfe^61^.

### Detecting signature of selection

To identify gene wide selection the “Branch-site Unrestricted Statistical Test for Episodic Diversification (BUSTED)”^62,63^ approach was used from the Datamonkey webserver^64–66^ using a p-value of < 0.05 as the threshold for significant results.

As the gene wide model can mask specific sites under different selection pressures, the site based approaches ‘Fast, Unconstrained Bayesian AppRoximation (FUBAR)’^67^ and ‘Mixed Effects Model of Evolution (MEME)’^68^ were used from the Datamonkey webserver^64–66^. FUBAR can identify amino acid sites under pervasive positive or negative selection. This model utilises a Bayesian algorithm and the selection signature of a site is determined using posterior probabilities rather than a p-value. A posterior probability of > 0.9 strongly suggests positive selection, however in the current study only sites with a posterior probability of > 0.95 were considered. The stricter threshold was used to account for the large evolutionary distance when analysing the mammalian class as a whole. Since FUBAR only identifies selection on sites across all branches, another model, MEME was used to identify signatures of selection on single branches. A branch represents a single species or a group of genetically, highly similar species in the alignment. MEME analyses each site to identify the presence of positive or diversifying selection with a mixed effects maximum likelihood approach. This model accounts for the effect of negative selection calculated from other branches in the alignment which would typically mask the positive selection on a site on specific branches. MEME uses p-values to determine statistical significance. As with FUBAR a stricter threshold was used, accepting only sites with a p-value of 0.01. Bar plots were generated using ggplot2 (V3.5.0)^69^.

Functional domains of each gene were identified with the Simple Modular Architecture Research Tool^70^ by using the human sequences as reference templates. The orders Dermoptera, Eulipotyphla, Lagomorpha, Pholidota and Scadentia were not assessed as taxa due to low sample sizes (Table 2) and the inability to be effectively grouped into a higher taxon (i.e., Cingulata and Pilosa forming Xenarthra) without being masked by other orders with higher species number. Since marsupials present an interesting taxon to study (with known high cancer rates^71–73^) and as there were seven marsupial genomes available at time of analysis, the minimum number of species per taxa was limited to seven in all the downstream analyses.

A logistic regression model was run in R studio (V. R 3.6.2) (RStudio Team 2020), to test whether the number of genomes analysed per taxa had any effect on the number of detected sites under selection by MEME and FUBAR compared to the number of species/taxa. A chi squared post hoc test using (Lme4 V1.1-30^74^, chisq.posthoc.test^75^) was then performed to identify taxa with more or less sites than expected based on the number of species in the taxa.

## Results

### Phylogenetic analysis

**Figure 1:**
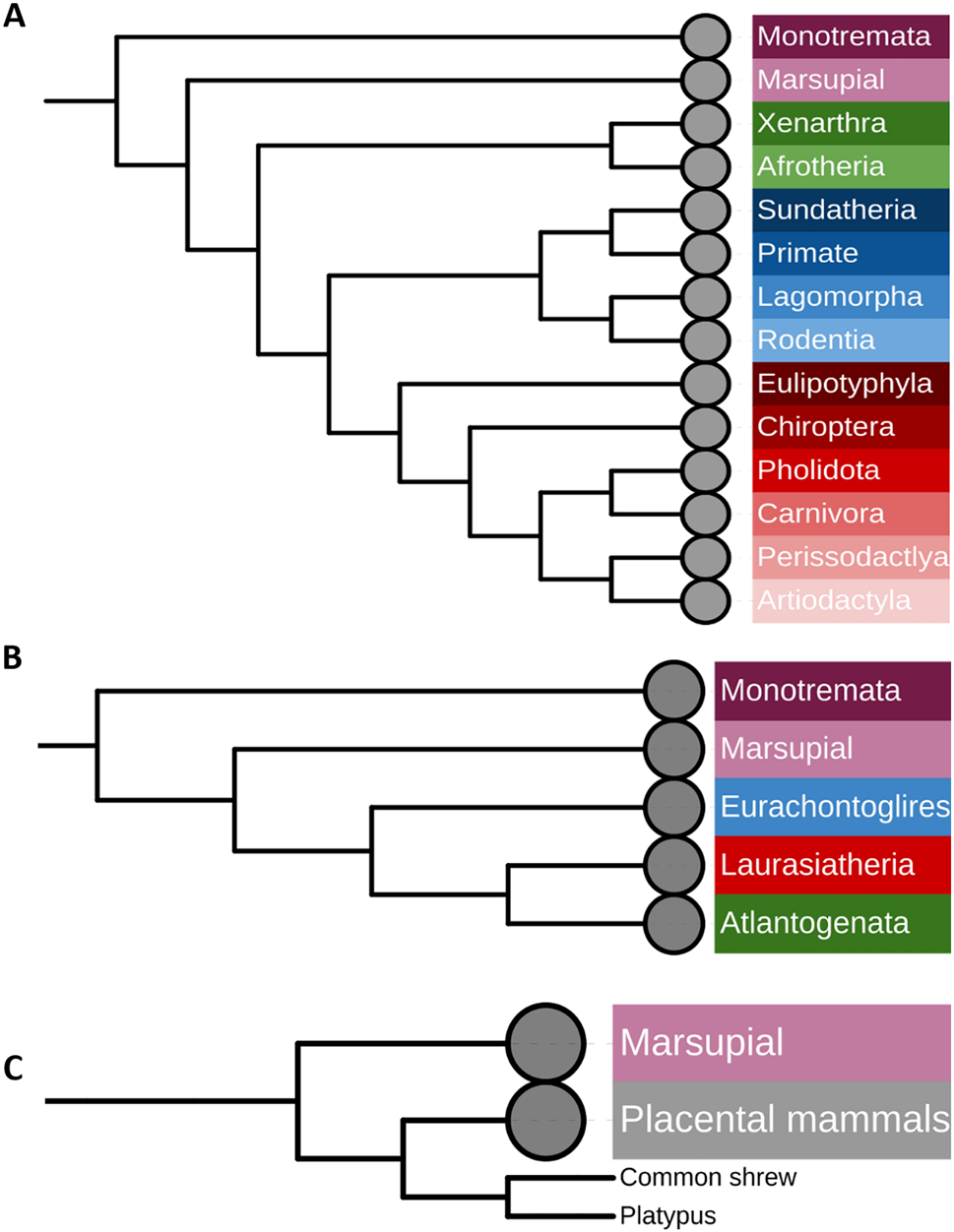
Phylogenetic reconstructions of mammalian species evolution, as well as the evolution of genes of interest. (**A**) Phylogenetic tree illustrating the current consensus for the phylogenetic relationships of mammalian species (derived from: Upham et al.^107^). Trees B and C were constructed in MEGA-X^58^. Phylogenetic trees were modelled using Kimura 2-parameter model^59^, and maximum likelihood analyses were run with 500 bootstrap replications and a bootstrap cut off value of 70 on the final tree. Nearest neighbour joining tree inference was used. The phylogenetic trees were visualised using iTol V5^60^. (**B**) The phylogenetic analysis of mammalian Casp8 showed Euarchontoglires form an ancestral relationship to Laurasiatheria in the place of Atlantogenata contradictory to the evolution of the taxa. The order compositions of these taxa are represented in (**A**) as; Euarchontoglires in shades of blue, Laurasiatheria shades of red and Atlantogenata shades of green. (**C**) The phylogenetic analysis of mammalian GRB2 sequences showed the platypus forming a monophyletic group with Eutherians contradicting the ancestral relationship of the platypus to Eutherians and Metatherians.

A phylogenetic analysis was conducted using a comparison with the currently accepted evolutionary relationship of mammals (Fig. 1A), to establish whether the evolution of cancer associated genes followed or diverged from, the species evolution. In general, the genes followed the same pattern of evolution as the species, while the most interesting patterns were found in Casp8 and GRB2 (see Supplementary S1). The evolution of Casp8 shows that the Euarchontoglires, not the Atlantogenata, are the root ancestor of the Eutherians (Fig. 1B). For GRB2 (Fig. 1C), the platypus (*Ornithorhynchus anatinus*), which had been presenting as the root for mammals in all other gene trees, forms a monophyletic group with Eutherians while Marsupials maintained an ancestral relationship. When analysing the exact position of the platypus, it forms a monophyletic relationship with the common shrew (*Sorex araneus*). Following reconstruction of the phylogenetic tree excluding the common shrew the same pattern was observed, and the platypus remained with the Eutherians. Apart from the unusual evolution of GRB2 in the platypus, most orders exhibited soft polytomy with the exact divergence order of many species being indistinguishable.

### Detecting gene wide selection

Oncogenes appear to be under the lowest amount of episodic diversifying selection in most taxa (Table 3). Tumour suppressor genes were under episodic positive selection in most taxa, although this pattern was mostly driven by selection in Casp8 and BRCA2. With LITAF having three taxa showing positive selection and BRCA2 showing diversifying selection in eight of the nine taxa. A similar pattern was seen amongst immune genes, with CD274 and B2M being under diversifying selection in most taxa, and IL2 showing diversifying selection in the same three taxa as LITAF.

**Table 3:**
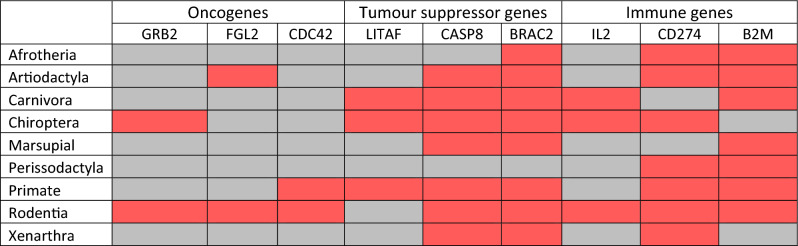
Episodic diversifying selection in mammalian cancer associated genes.

The Branch-site Unrestricted Statistical Test for Episodic Diversification (BUSTED) approach was used to identify episodic diversifying selection. Genes under diversifying selection are highlighted in red, those that are not, are highlighted in grey. A p-value of < 0.05 was the threshold for significant results.

Rodentia had the highest number of genes under episodic positive selection, with all studied genes except LITAF under selection. With Chiroptera having the second highest number of genes under selection. The taxa with the fewest genes under selection were Afrotheria, Marsupials, Perissodactyla and Xenarthra. Notably, the aforementioned taxa all contained fewer than ten genomes in the analyses.

### Detecting site specific selection

As gene wide models of selection can obscure selection site specific selection, a site-based analysis was conducted using the FUBAR and MEME algorithms^67,68^. Most genes showed signatures of predominantly negative selection. The negative selection was often scattered throughout the gene with clusters outside functional domains. For CDC42, all amino acids were under either negative selection or were neutral across mammals (Figs. 2, 3 and Supplementary S1, Figs. 1–18).

**Figure 2:**
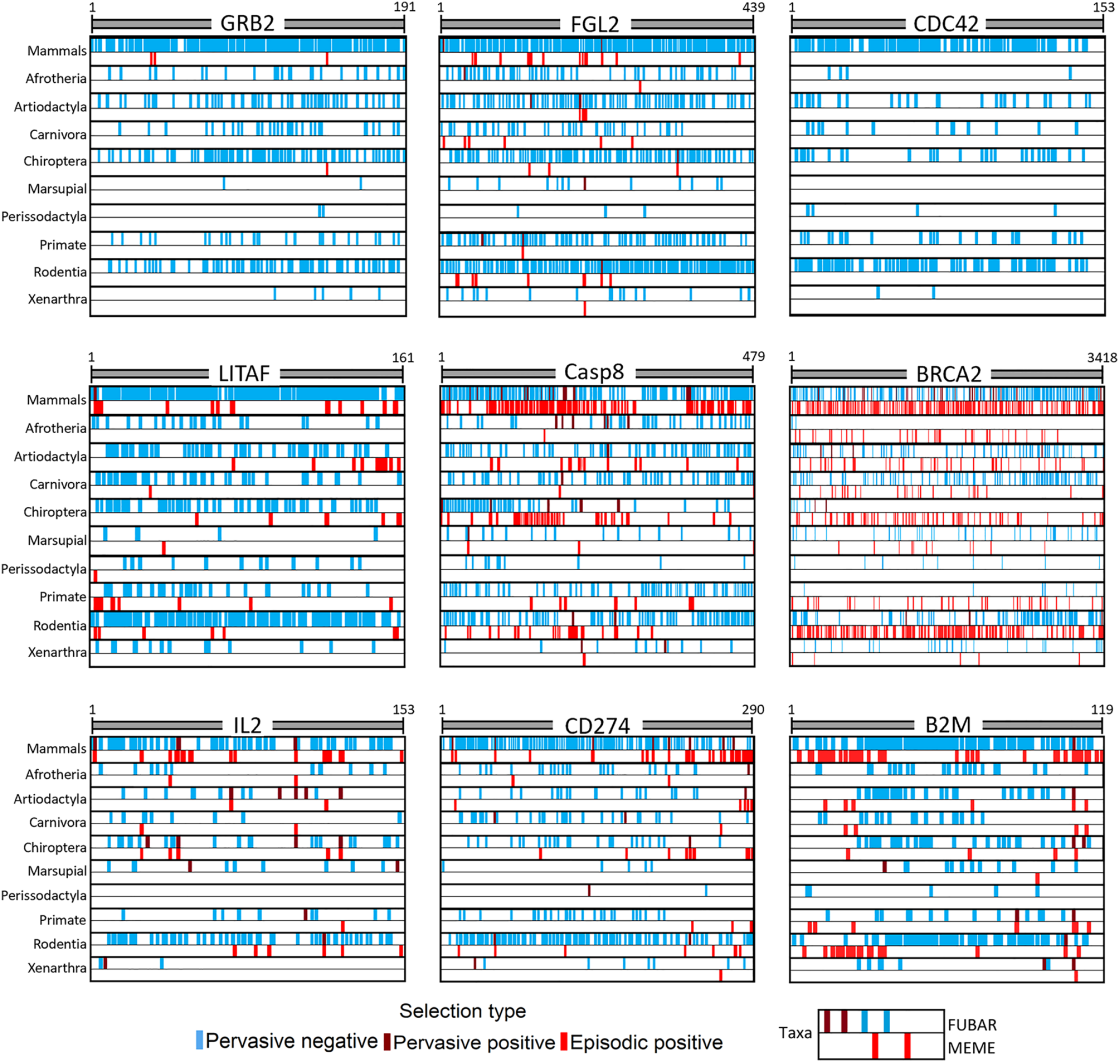
Signatures of selection across amino acid sites of cancer associated genes. Detection of pervasive selection using ‘Fast, Unconstrained Bayesian AppRoximation (FUBAR)’^67^ and episodic positive selection using the ‘Mixed Effects Model of Evolution (MEME)’^68^ was conducted on the nine cancer associated genes. The tests were run across different Mammalian taxa, ranging from class to order. Highlighted are gene regions under selection, blude indicates negative and red indicates positive selection. Numbers on top of each figure indicate gene length based on number of amino acid residues.

**Figure 3:**
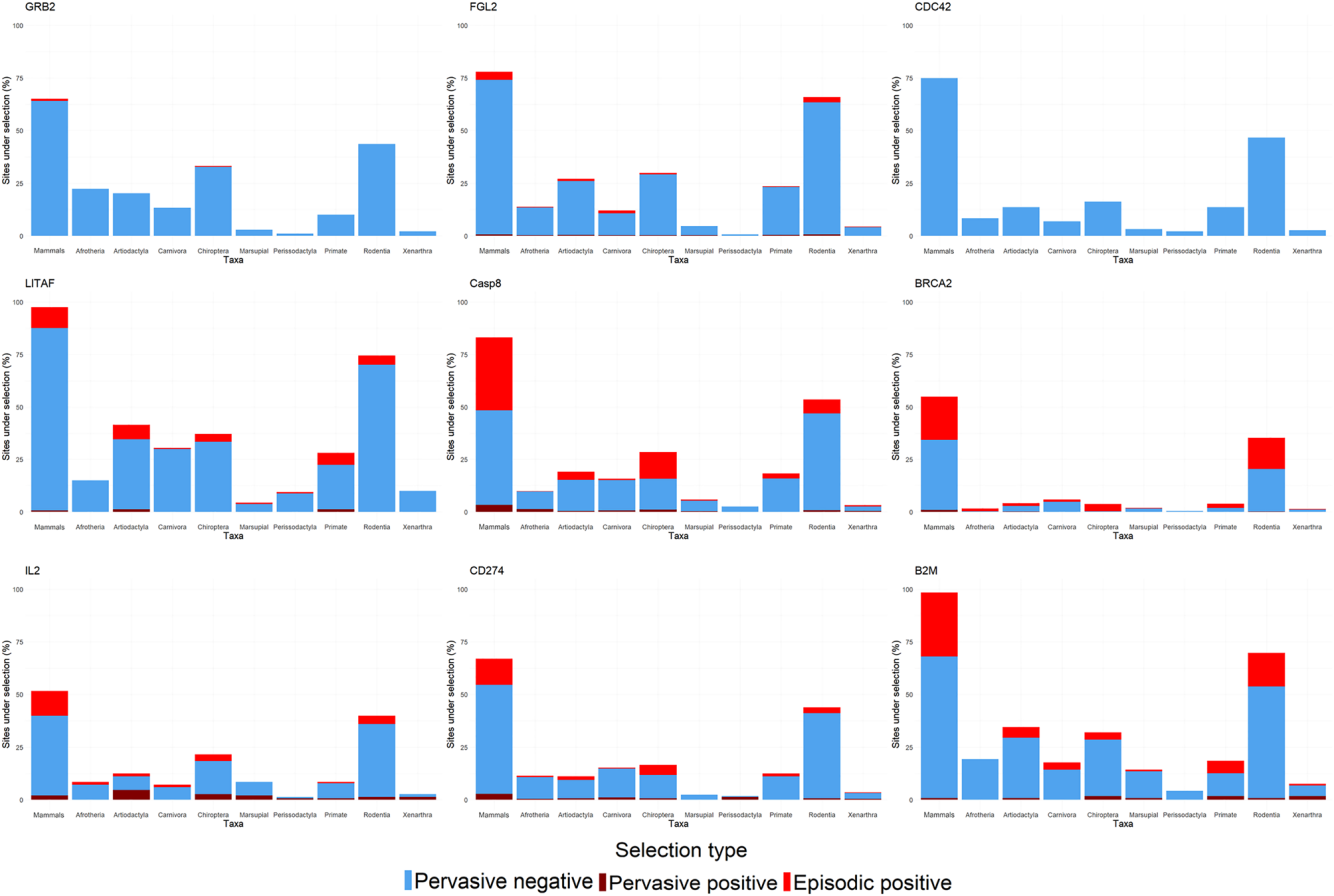
Percentage of sites under varying types of selection in cancer associated genes. Bar plots show the percentage of sites under selection in each taxon for the nine cancer associated genes. The sites are comprised of pervasive positive and pervasive negative selection as identified by ‘Fast, Unconstrained Bayesian AppRoximation (FUBAR)’^67^ and episodic positive selection identified by ‘Mixed Effects Model of Evolution (MEME)’^68^.

**Figure 4:**
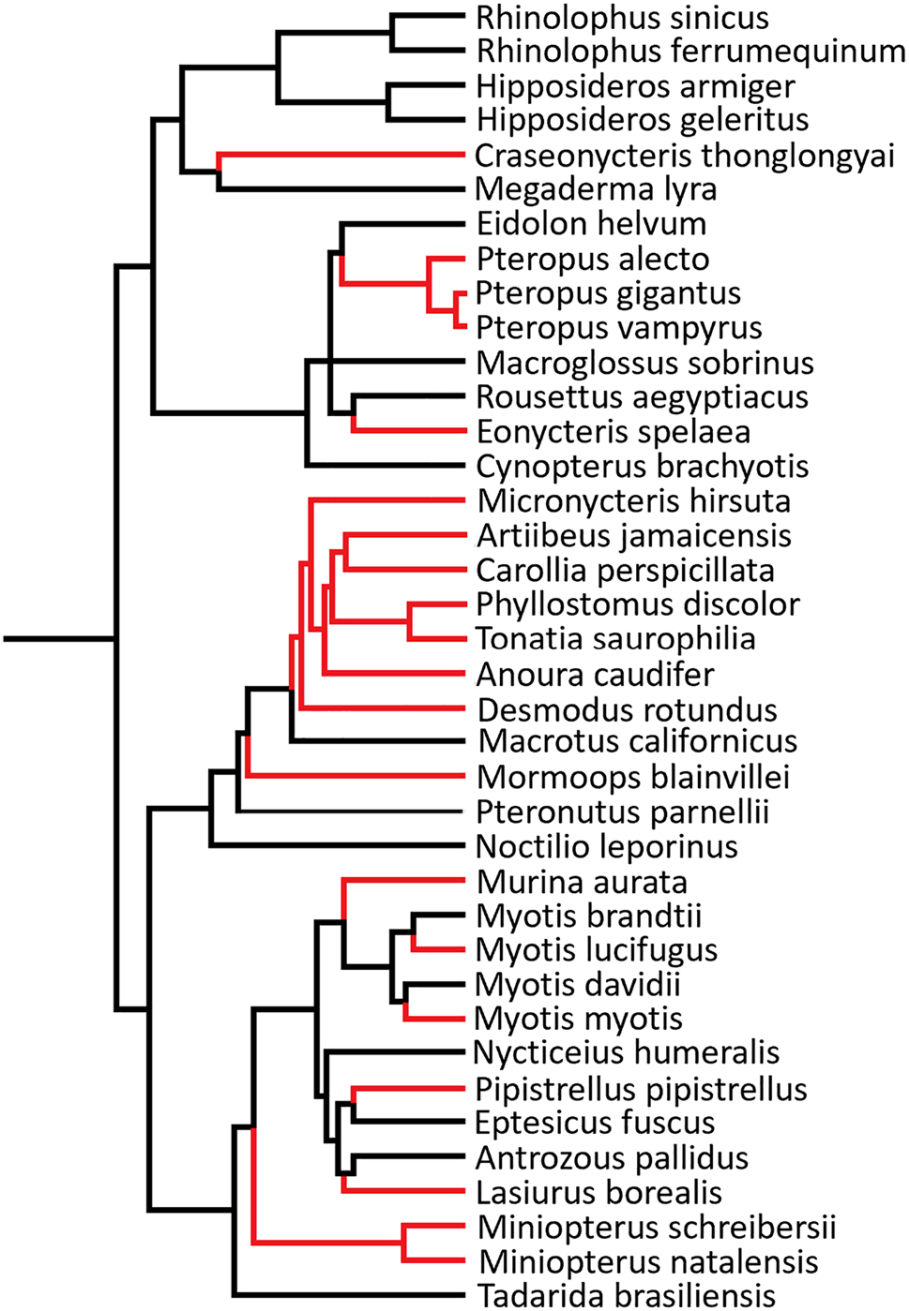
Chiropteran species showing positive selection in the DED2 domain of CASP8. The tree shows the phylogenetic relationship of order Chiroptera. Red branches indicate species where positive selection was detected in the second DED (DED2) domain of Casp8, using the ‘mixed effects model of evolution (MEME)’^68^.

Most amino acids in Casp8’s first DED domain (DED1) were under negative selection in mammals. In contrast, the second DED domain (DED2), while exhibiting some negative selection, had more amino acid sites under episodic positive selection. The contrasting pattern is likely driven by Chiropterans, while the DED1 domain is under mostly negative selection in this taxon, the DED2 domain is under mostly positive selection. When determining which species are driving this result within Chiroptera, there appears to be no phylogenetic pattern. Species across the taxa show high amounts of positive selection in DED2 (Fig. 4). The region between the DED2 and Casc domains also appears to be under positive selection among the Chiropterans, Artiodactyla and Rodents. Signatures of both positive and negative selection were observed in the Casc domain. Functionally important Cysteine residues: C309, C312, C331, C345, C433 were all under negative selection across mammals and no positive selection was observed in any taxa for these sites. The Chiropterans had the highest proportion of episodic positive selection with 13% of sites under positive selection, followed by the Rodentia with 7% (Fig. 3).

Most amino acid sites were under negative selection in the remaining genes. In CD274, episodic positive selection clustered within the C terminal end of the protein, while minimal positive selection occurred in the functional domains. This pattern is again primarily driven by the orders Chiroptera and Artiodactyla. When analysing all mammals together, only eight sites were under both pervasive and episodic positive selection. The lowest percentage (38%) of negative selection amongst all genes was observed in IL2. Interestingly, no amino acid sites in IL2 were under any type of selection in Perissodactyla, and no sites were observed under episodic positive selection in Marsupials and Xenarthra. A few amino acids were detected to be under pervasive positive selection in the N terminal region deemed functionally important and there was no positive selection in the functionally important C-terminal region. Across the taxa, the most sites under negative selection were observed in the Rodentia with 35% of amino acids under purifying selection, followed by the Chiroptera with 16%. While amino acid sites 58 and 105 of IL2 are functionally important cysteine residues, negative selection was only detected at site 58, and no positive selection was seen at either of the sites.

The highest proportion (87%) of negative selection was observed in LITAF across all genes, and only a single amino acid site was under pervasive positive selection across all mammals in this gene. Episodic positive selection was observed sporadically across the gene, and approximately the same proportion of amino acid sites were under episodic positive selection (10%) as in the genes CD274 and IL2, which was 12% in both. Similar to other genes, most amino acid sites were under negative selection in FGL2, a pattern driven by Rodentia in this gene, where 62% of sites were under negative selection with the Artiodactyla being the next highest at 26%. Most sites were under episodic positive selection outside the functional domains, a pattern mostly driven by Artiodactyla.

For taxa with low sample size, few amino acid sites appeared to be under any type of selection in the taxa with low sample sizes (Afrotheria, Perissodactyla and Marsupial) by this detection method. The low number of sites under selection across all genes for these three taxa raised concerns about low sample sizes affecting the results of FUBAR and MEME analyses. Thus, a logistic regression analysis was conducted to determine whether the number of species within a taxa influenced the selection analyses results (Supplementary S1, Tables 1, 2, 3). In the regression analyses of the individual genes the slopes ranged from 0.016 (IL2) to 0.025 (LITAF). While these slopes were significant, they were very close to 0 and were not biologically relevant. A Chi-square posthoc test (Table 4) showed that Marsupials, Perissodactyls and Xenarthrans had fewer than expected sites under selection across all genes based on the number of species in those taxa. Even Afrotheria, which had comparable site selection result to orders like Carnivora, were not yielding high numbers of sites. The expected sites were 11 times lower than predicted for Perissodactyls in BRCA2, and 35 times higher in Rodents for both groups represented the extremes based on the number of species across all genes (Table 4).

**Table 4:**
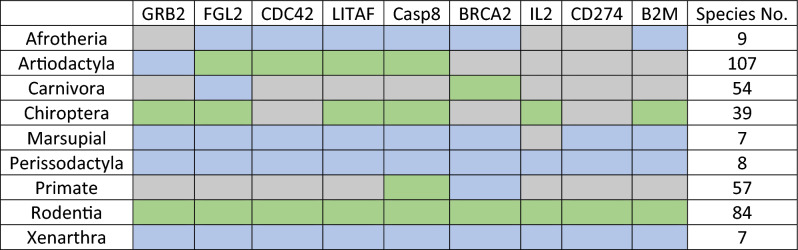
Interaction between genome sample size and number of sites under selection.

Results of a chi square posthoc analysis to determine if number of species in each mammal taxa influenced the site-specific selection analyses Fubar and MEME. The number of sites was acquired by combining the total number of sites detected by both the ‘Fast, Unconstrained Bayesian AppRoximation for Inferring Selection (FUBAR)’^67^ and the ‘Mixed effects model of evolution (MEME)’^68^ programs. Negative values (blue) are lower than expected, positive values (green) are higher than expected and values in grey are non-significant (p-values 0.05).

## Discussion

Cancer is a disease that appeared at the dawn of multicellularity and has been present in multicellular species for millions of years^1^. In the fight to contain cells going rogue, species have evolved various tumour suppression mechanisms^14^, however not all species are as capable of preventing oncogenesis as others^71,76^. As the impact of cancer on survival and reproduction is not the same across species, evolutionary pressure for adaptations to cancer vary. Comparative genomics is a powerful method to detect selection pressures in cancer associated genes and to shed light on the evolutionary history of cancer resistance and susceptibly among taxa. This study expanded on previous comparative genomics studies in mammals utilising a comprehensive comparison of publicly available mammalian gene sequences and increasing the number of species analysed, including the number of species from previously underrepresented marsupials. Marsupials are of particular interest in cancer biology due to their shorter lifespans compared to Eutherians^77^, and their relatively high susceptibility to cancer (especially the Dasyurids^72,73,78^). We assessed gene wide and site-specific selection pressures in the context of trends across categories of cancer associated genes, i.e., oncogenes (GRB2, FGL2 and CDC42), tumour suppressor genes (LITAF, Casp8 and BRCA2) and immune genes (IL2, CD274 and B2M), and extended the analyses to mammalian taxa on the order, super order and infraclass level.

### Phylogenetic reconstruction

The phylogenetic analysis revealed that for most species the evolution of cancer associated genes follows the species’ evolution, and overall, no specific clustering of species was observed in the evolutionary history of the studied genes in relation to their involvement with oncogenesis. This is not surprising, as the genes have fundamental roles in cellular, organismal, and physiological functions that could constrain and dictate their evolution. Additionally, the method used in this study accounts for sequence characteristics across whole sequences when establishing relationships and adaptations. So, small nucleotide variations, if present, may remain undetected.

The reconstructions of the evolutionary relationship of the GRB2 and CASP8 genes yielded unexpected findings. The platypus GRB2 gene formed a monophyletic group with the common shrew (*Sorex araneus*) along the branch with the other Eutherian (placental) mammals, no longer retaining its ancestral relationship where it diverged before marsupials. As all other mammals form a monophyletic group excluding the Marsupials, this suggests that there may have been novel nucleotide changes in marsupial GRB2. Alternatively, the platypus and the other mammals may have maintained a conserved sequence over evolutionary time, and it is marsupials that evolved a novel sequence. It is unclear why this relationship has occurred. GRB2 encodes an adapter protein central to fundamental signal transduction/cell communication^44^, and it is difficult to determine what may have caused the mutations in this species. Incorporating the sequences of other Monotremes may help further contextualise the findings, although the power to understand the mutational history is constrained with only five species of Monotremes in existence^79^, four of which belong the family of echidnas (Tachyglossidae). Functional analyses of the GRB2 proteins from the different species might reveal the importance of nucleotide changes of these gene in Mammals.

The gene tree of CASP8 showed similar phylogenetic relationships to the species tree, but it also demonstrated divergence in the relationships of the eutherian taxa Atlantogenata, Euarchontoglires and Laurasiatheria. Atlantogenata formed a monophyletic group with Laurasiatheria in the gene tree. These patterns may have arisen due to convergent and rapid evolution, that could have led to similarities between distantly related species or in large differences between closely related species. The rooting of the Eutherian tree has been an ongoing debate as it has been uncertain which taxa of Eutherians diverged first. Previously three theories were proposed: (i) either the entirety of the super order Atlantogenata (Xenarthrans and Afrotherians) was the root or that it was either the (ii) Xenarthrans or the (iii) Afrotherians that had diverged first^80^. Interestingly, the results obtained here contradict the basis of all of these theories by showing none of the groups to have diverged first. This result may be due to that a phylogenetic tree constructed based on the sequence alignment of a given gene represents the evolutionary history of the gene that may not align with the evolutionary history of the species (reviewed in^81^).

Despite the substantial variation in the multiple sequence alignments of each gene, our results revealed that most of the mammalian phylogenetic trees contained extensive polytomy. We predict that the observed polytomy represents diverse rates of speciation events and varying evolutionary distances between species within the studied groups, and such, it impacts the complexity of the phylogenetic inference in our trees.

### Detection of gene wide selection

Overall, the signature of selection associated with each type of cancer gene observed in our study supported the findings of previous research^26,27,32^. Oncogenes were the most conserved, while tumour suppressor and immune genes both showed higher amounts of episodic diversifying selection. The signatures of episodic diversifying selection on immune genes across taxa was expected due to their role in recognising and responding to a plethora of pathogens and environmental challenges^29,43,82^. Given that TSGs are typically highly conserved^32^ and immune genes show high variation^29,43^, it would be expected that diversifying selection would be less prevalent in TSGs. However, episodic diversifying selection was observed in two of the studied TSGs, CASP8 and BRAC2, while LITAF was more conserved across the mammalian taxa. The discrepancy in selection patterns across the TSGs may be explained by their involvement in different aspects of tumour suppression. The members of CASP family are sequentially activated during the execution-phase of cell apoptosis and CASP8 is a mediator of death receptor signalling^47^. In contrast, BRAC2 is involved with a different aspect of cell proliferation, notably double-strand break repair and/or homologous recombination during cell division^48^. LITAF encodes a DNA-binding protein, lipopolysaccharide-induced TNF-alpha factor, that is implicated in the p53-induced apoptotic pathway. The transcription of LITAF is induced by the tumour suppressor p53, that is activated in response to many stress stimuli (e.g. oncogenes activation and DNA damage), and once activated, it directly regulates the transcription of ~ 500 genes and controls diverse cellular processes^83^. Whether the high conservation of LITAF across mammalian taxa observed in our study is due to its association with the central gate keeper P53, remains to be answered. De- or over- activation of these genes in vitro in cells from and across different mammalian taxa (with different mutations in TSGs), might be able to decipher how they function and change in different species.

Furthermore, Casp8, BRCA2, CD274 and B2M were under diversifying selection in most taxa. Casp8, CD274 and B2M’s involvement in immunity^84,85^, was likely driving positive selection in these genes. Interestingly, BRCA2, a key gene involved with DNA repair as well as with embryogenesis^86^ showed signatures of episodic selection in 8 out of the 9 taxonomic groups studied here. A potential explanation for this conundrum (as high conservancy of this gene could be expected due to its critical role in cellular functions) could be its large size (10,254 bp nucleotides) and involvement of its different domains with multiple functions^87^. Under our site-specific selection analyses, we indeed detected different types of selections in different domains of this gene. It is also possible, that despite of its key caretaker function, this gene might be under varying selection pressures in taxa with different reproductive strategies, and hence the observed gene wide episodic diversifying selection on this gene.

Interestingly, a previous study found positive significant correlation between single nucleotide polymorphism (SNPs) and gene and transcript lengths^88^. However, when looking at the relationship between evolution and transcript length based on dN/dS ratios across three species (mouse, gorilla, chimpanzee), Lopes et al.^88^ found longer genes being more conserved than shorter ones. In our study BRAC2 was the longest gene (Fig. 2) and presented with episodic diversifying selection in all but one (Perissodactyla) mammalian group studied by us. Our results may indicate that longer genes may not evolve under stronger evolutionary constraints, as suggested by previous studies^88^. However, it is important to point out that the two studies, Lopes et al.^88^ and us, used different approaches: large gene sets from three species vs few genes across 386 Mammalian species, respectively. Repeating the analyses of Lopes et al.^88^ on a larger dataset may be necessary to expand on our knowledge on how older and longer genes evolve compared to short, most recently emerged gene families.

Here we propose that function and interactions (e.g., with pathogens) may be more important selection forces than gene size. For example, in CD274 (290 amino acids), seven of the nine taxa were under diversifying selection, where in CDC42, which is similar in size (274 amino acids), only had two taxa showing signs of diversifying selection. Profound understanding of the specific roles of various regions of these genes across different species would be needed to fully understand how selection shapes their evolution.

Oncogenes, CDC42, FGL2 and GRB2 were found to be highly conserved. Due to their vital role in multiple signalling pathways and maintaining cellular homeostasis^89,90^, it is expected that strong negative selection will ensure the removal of deleterious mutations from these genes. In addition, as relatively few mutations can cause proto-oncogenes to become oncogenic, negative selection is anticipated to eliminate any fitness limiting mutations. For GRB2 the only taxa that showed diversifying selection were Chiroptera and Rodentia, which had shown some of the most genetic variability in the analysis.

As found in the TSGs, taxa with lower sample sizes had fewer genes showing diversifying selection, indicating that comparative analysis of small and underrepresented taxa may be limited.

### Detecting site specific selection

Varying strengths of negative selection was detected across the studied genes. The site-based analysis of Casp8 revealed that the two Death Effector Domains (DED) in Chiropterans (bats) show contradictory results, with the first DED domain being under negative and the second domain being under positive selection in the taxa. The tandem DED domains (tDEDs) are responsible for Casp8 recruitment^91^, with the domains binding to signalling molecules such as FADD in the induction of apoptosis^92^. The tDEDs are used in Casp8 recruitment for multiple other signalling pathways, including ones relating to inflammation^93,94^. This is interesting, as longevity and suppressed inflammation are notable traits in bats^95^. Many species of bats are known to be reservoirs for zoonotic diseases, facilitated by bats with less extreme inflammatory responses compared to humans^96,97^. This helps negate the damaging secondary effects of an immune response that often cause morbidity/mortality, but it also allows pathogens to replicate and subsequently spread to other organisms. It is likely that the selection pressures on tDEDs are shaped by pathogen exposure. Many pathogens interact with Casp8 and viruses (e.g. Adenovirus) have homologues that replicate the function of the Casp8 inhibitor, cFLIP^98^. As cFLIP interacts with Casp8 through the second DED domain, variation would need to occur in the second DED domain if the evolutionary response was to pathogens.

To avoid the detection of false positives, a known shortcoming of MEME^68^, a more conservative positive selection threshold was used in this study. Therefore, it is unlikely that the observed patterns have arisen due to the approach used. Nevertheless, it should also be considered that the observed positive selection could simply indicate that residues at these sites are not as functionally important, resulting in reduced negative selection and more genetic variability in bats. As the positive selection was not observed as branch specific or consistently across all Chiropterans and some species did not show any positive selection in DED2 (Figs. 2 and 4), these further indicate that the observed positive selection is influenced by pathogens individual species are exposed to. These findings would need to be followed up by analysing the potential structural and functional changes that arise in Casp8 to determine biochemical significance of the observed nuclear and amino acid patterns.

The most distinct patterns in BRCA2 were found in Rodentia. While much of the gene was showing positive selection, there was evidence of negative selection in the binding domains (DBD region) (Fig. 2 and supplementary S1, Fig. 12). This aligns with previous research showing high variation in the evolution of this gene between eukaryotic species as well as the evidence of select regions being conserved^99^. When looking at sites with known pathogenic mutations in BRCA2; W31C, K2630A, D2723 and Y3308^100^, none of the sites were under positive selection in either the FUBAR or MEME analysis for any taxa. In fact, both the amino acid sites W31 and W2626 (know for a W2626C cancer mutation^101^) were 100% conserved in their nucleotide sequence across all mammalian species. This indicates that despite variation across other sections of the gene, there are key sites that are highly conserved and thus are most likely important for mammalian health and development.

In LITAF, the mutation (G112S) known to be associated the Charcot-Marie-Tooth disease, is present in the genome of Sowerby's beaked whale (*Mesoplodon bidens*). No positive selection was detected for this site in any taxa. Given that this is a genetic disease found only in humans, the consequences of this mutation may have less of an impact in the whale species. The comparison of cancer associated genes in hominoids found cancer causing mutation in non-human primates that were a part of the germline^27^ and may not cause cancer in these species. Protein–protein interactions and changes in the genetic sequences of other genes would potentially have alternative mutational pathways to diseases in other species^27,36,37^, adding a layer of complexity to interspecies genomic comparisons.

It’s also important to take in account variation of evolution within tumour sub-populations^102^. As mutational pathways would also vary with the different challenges present in the tumour microenvironment and a cancer’s strategy can differ in response to this environment^103^. For example, in the tumours of the Devil Facial Tumour Disease, B2M is downregulated in order to evade detection by the immune system^104^. This immune evasion is extremely effective, with minimal immune cell infiltration into the tumours. As a result, the primary tumours have reduced pressure to express CD274 to suppress T-cells that would typically target them, and do not express the gene above the range of healthy tissue^105^.

The question of sample size was raised by the site-specific analysis. In marsupials, Perissodactyls and Xenarthrans, three taxa with low species numbers (N   < 10) appeared to have fewer sites under any type of selection compared to other taxa. The MEME model is known to be more conservative for lower sample sizes^68^ and the stricter threshold for the identification of positive selection used in the study would have confounded the identification of fewer sites. However, Afrotheria with nine available genomes, appeared to have similar numbers of sites under selection as the Carnivorans (N = 54), suggesting there was no consistent association between species number and the number of sites under selection.

Furthermore, a logistic regression model indicated that there was no significant association between the number of sequences and the number of sites detected. However, when looking at the expected and observed number of sites under selection for each taxon, Afrotheria, Marsupials, Perissodactyla and Xenarthra had lower than expected values for all genes. Additionally, Artiodactyls, Carnivorans and Primates all had lower than expected values for half of the genes, whereas Chiroptera and Rodentia always had higher than expected. Remarkably Rodentia ranged between 7 and 35 times higher than expected. It is possible that Rodentia is potentially skewing the perception of what should be expected from the site-specific analysis.

## Conclusion

This study provides an extensive investigation into the evolution of nine cancer associated genes in mammals. It has greatly expanded upon the species used and conducted one of the most representative analyses of marsupials and mammals in the field of comparative oncology.

The analysis showed that although there is genetic variation over evolutionary time, the evolution of cancer associated genes in mammals primarily follows the phylogenetic relationships of their species. The lack of major changes in the genes can be attributed to their roles in fundamental and essential physiological functions. Most notably, the study identified contrasting selection pressure on the two DED domains of Casp8 in bats (Chiroptera) highlighting this pathway as interest for future research into cancer susceptibility and resistance in mammals. Episodic diversifying selection was observed in the key caretaker gene, BRCA2 showing that detailed understanding of the specific roles of various regions of BRCA2 across different species is still needed. Further studies investigating a broader set of genes^106^ would be necessary to decipher the exact mechanisms driving the evolution of cancer associated genes.

### Supplementary Information

Supplementary Information 1.

Supplementary Information 2.

## Data Availability

Data will be made publicly available on Dryad upon acceptance of the manuscript.
