# Supplementary material for: The molecular evolution of cancer associated genes in mammals
Source: Sci Rep. 2024 May 22;14:11650. doi: 10.1038/s41598-024-62425-0 (PMC11109183; doi:10.1038/s41598-024-62425-0)
Supplement: Supplementary file 1 — Supplementary Information 1. [file 41598_2024_62425_MOESM1_ESM.pdf]

Supplementary Material

The evolution of cancer associated genes across Mammals

Nick MacDonald, Nynke Raven, Wendy Diep, Samantha Evans, Senuri Pannipitiya, Georgina Bramwell, Caitlin Vanbeek, Frédéric Thomas, Tracey Russell, Antoine Dujon, Marina Telonis-Scott, Beata Ujvari

Table of Figures

Supplementary figure 1. Phylogenetic reconstructions of Mammalian cancer associated genes......... 11
Supplementary figure 2. GRB2 signatures of selection 12
Supplementary figure 3. GRB2 functional domains 13
Supplementary figure 4. FGL2 Signatures of selection 14
Supplementary figure 5. FGL2 functional domains 15
Supplementary figure 6. CDC42 Signatures of selection 16
Supplementary figure 7. CDC42 Functional sites 17
Supplementary figure 8. LITAF Signatures of selection 18
Supplementary figure 9. LITAF functional domains 19
Supplementary figure 10. Casp8 Signatures of selection 20
Supplementary figure 11. Casp8 functional domains 21
Supplementary figure 12. BRCA2 Signatures of selection 22
Supplementary figure 13. BRCA2 functional domains 23
Supplementary figure 14. IL2 Signatures of selection 24
Supplementary figure 15. IL2 functional domains 25
Supplementary figure 16. CD274 Signatures of selection 26
Supplementary figure 17. CD274 functional domains 27
Supplementary figure 18. B2M Signatures of selection 28
Supplementary figure 19. B2M functional domains 29

Table of tables

Supplementary table 1. Logistic regression model: effect of sample size on the detection of amino acid sites under selection 31
Supplementary table 2. Chi squared test; sample size on sites number of sites under selection 32
Supplementary table 3. Chi squared posthoc effect of sample size on sites number of sites under selection by sub- group 33

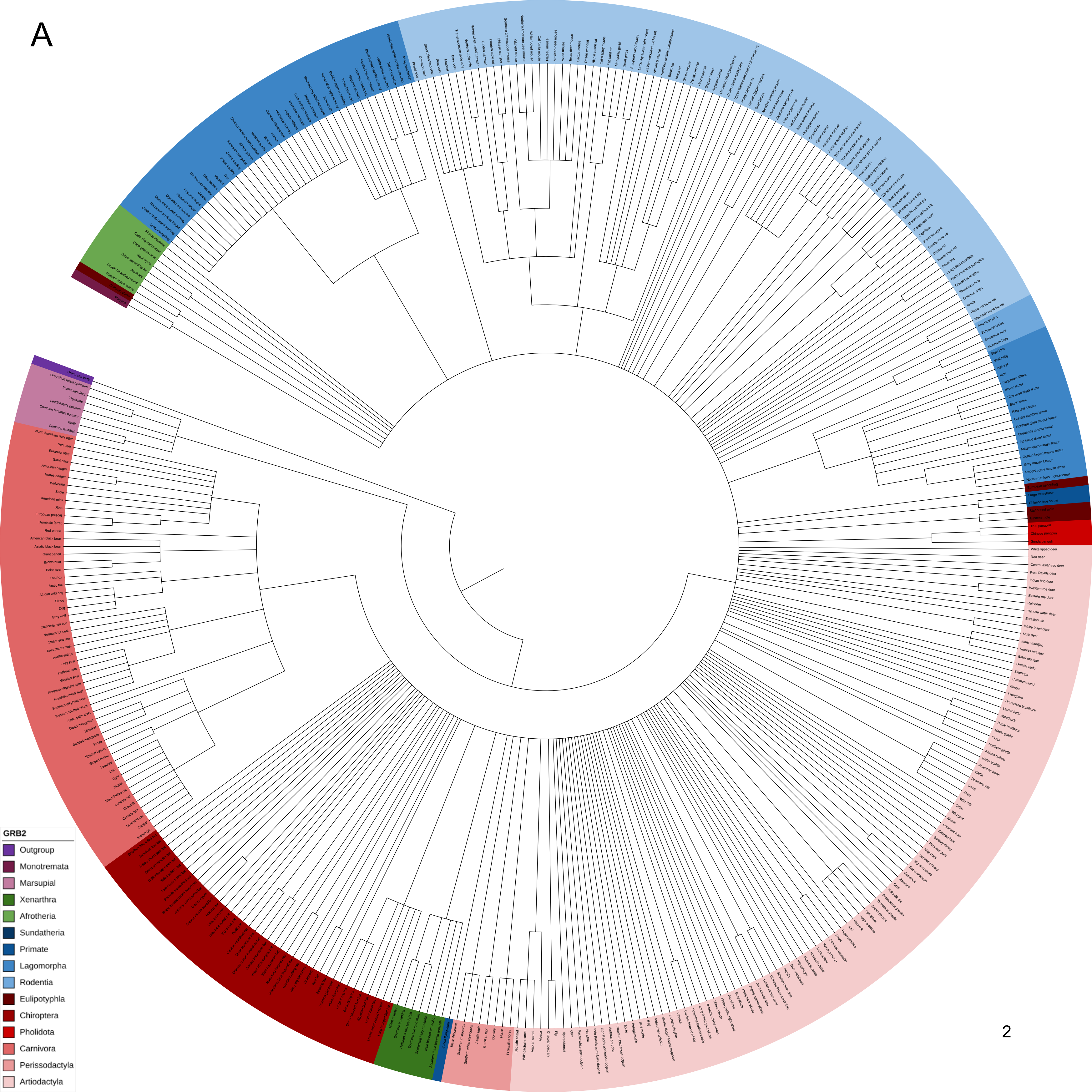
A
GRB2
Outgroup
Monotremata
Marsupial
Xenarthra
Afrotheria
Sundatheria
Primate
Lagomorpha
Rodentia
Eulipotyphla
Chiroptera
Pholidota
Carnivora
Perissodactyla
Artiodactyla

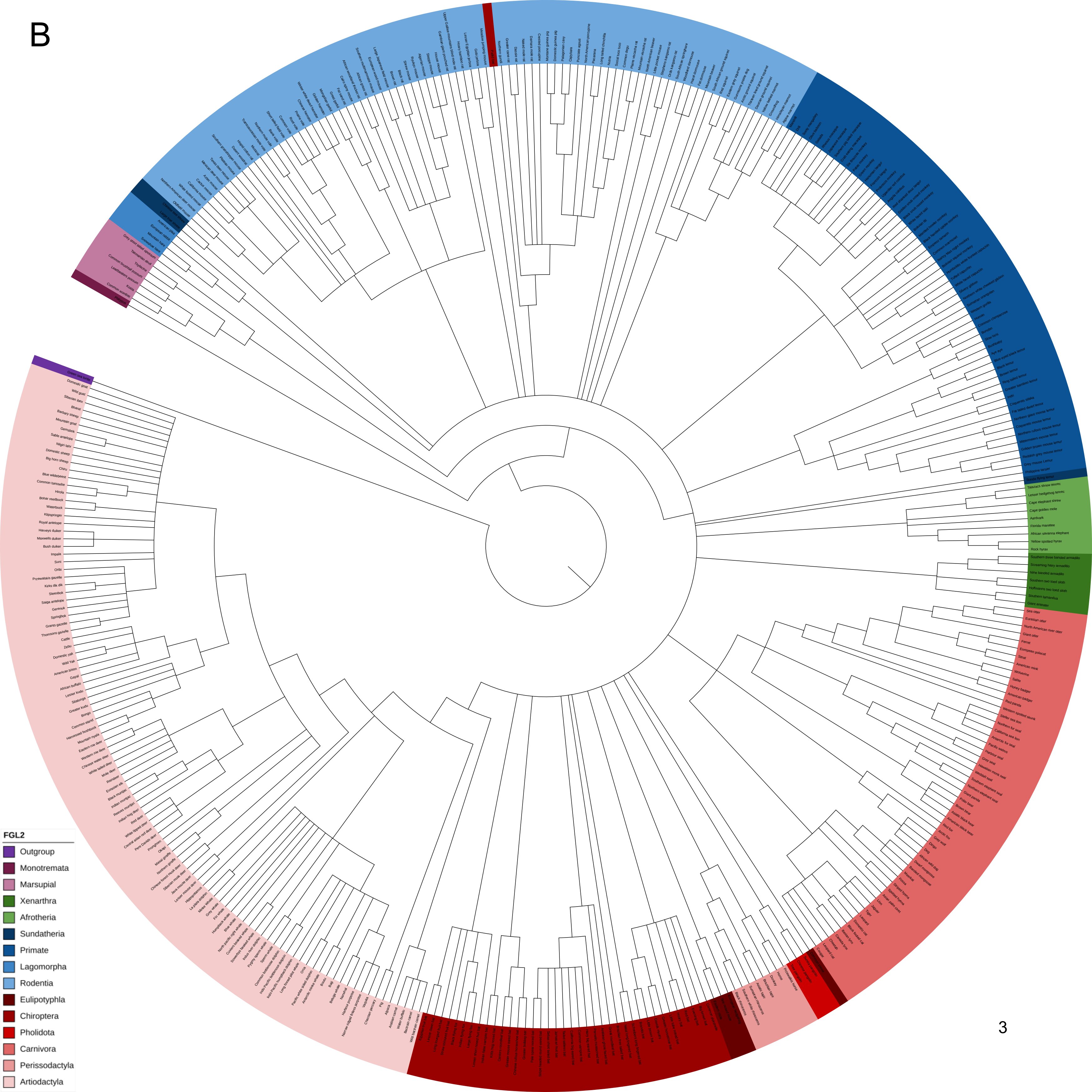
B
FGL2
Outgroup
Monotremata
Marsupial
Xenarthra
Afrotheria
Sundatheria
Primate
Lagomorpha
Rodentia
Eulipotyphla
Chiroptera
Pholidota
Carnivora
Perissodactyla
Artiodactyla

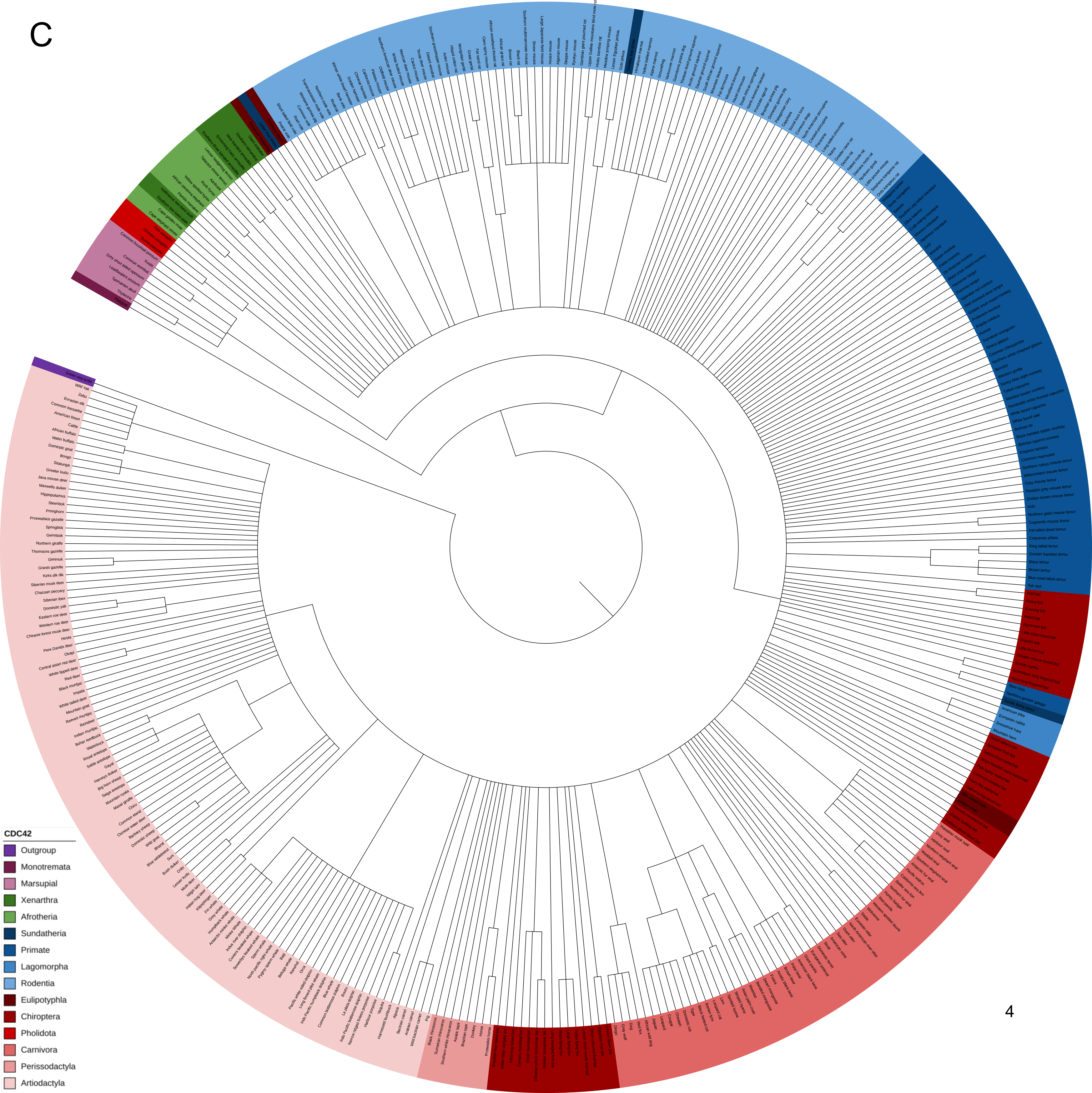
C
CDC42
Outgroup
Monotremata
Marsupial
Xenarthra
Afrotheria
Sundatheria
Primate
Lagomorpha
Rodentia
Eulipotyphla
Chiroptera
Pholidota
Carnivora
Perissodactyla
Artiodactyla

D

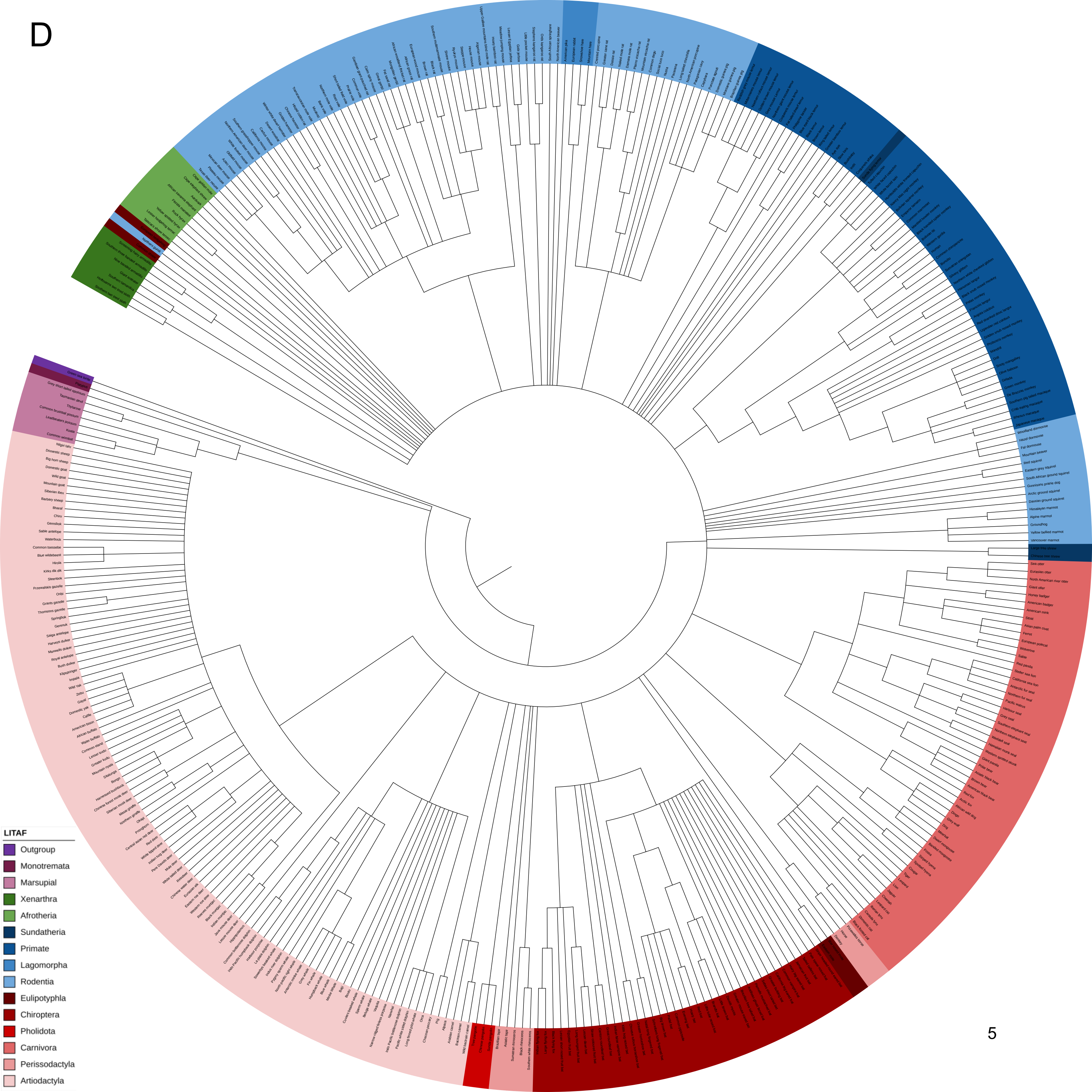

LITAF
Outgroup
Monotremata
Marsupial
Xenarthra
Afrotheria
Sundatheria
Primate
Lagomorpha
Rodentia
Eulipotyphla
Chiroptera
Pholidota
Carnivora
Perissodactyla
Artiodactyla

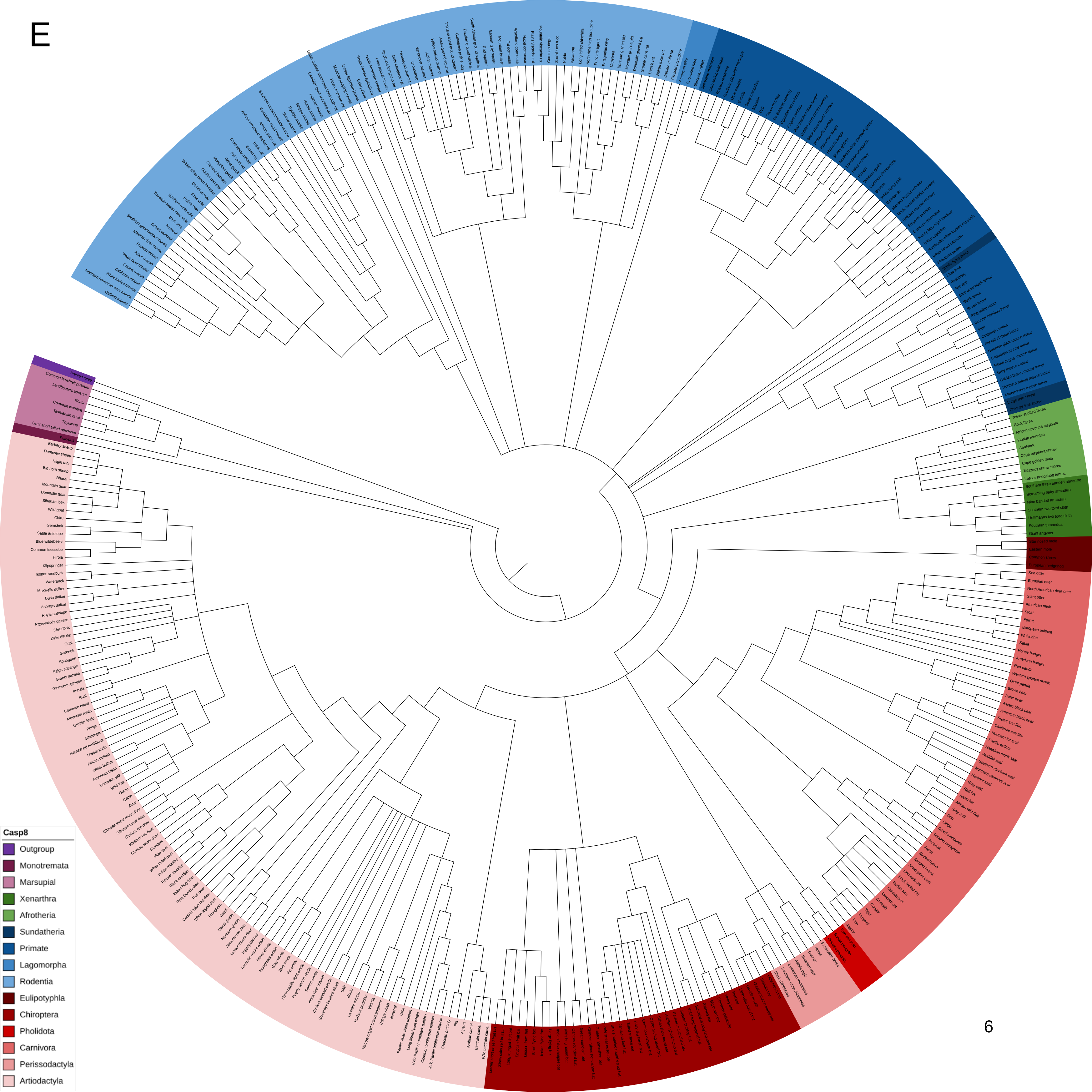
E
Casp8
Outgroup
Monotremata
Marsupial
Xenarthra
Afrotheria
Sundatheria
Primate
Lagomorpha
Rodentia
Eulipotyphla
Chiroptera
Pholidota
Carnivora
Perissodactyla
Artiodactyla

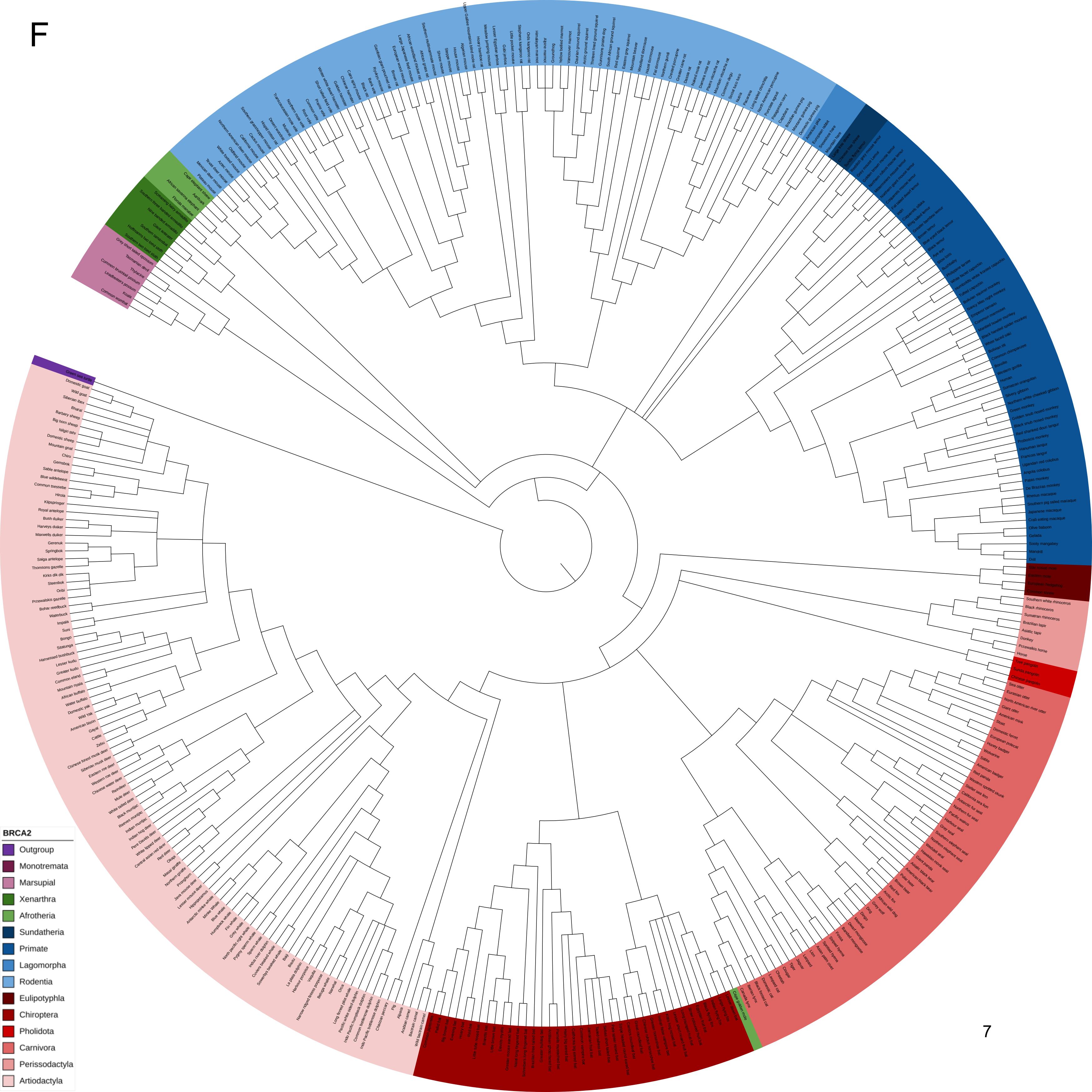
F
BRCA2
Outgroup
Monotremata
Marsupial
Xenarthra
Afrotheria
Sundatheria
Primate
Lagomorpha
Rodentia
Eulipotyphla
Chiroptera
Pholidota
Carnivora
Perissodactyla
Artiodactyla

G

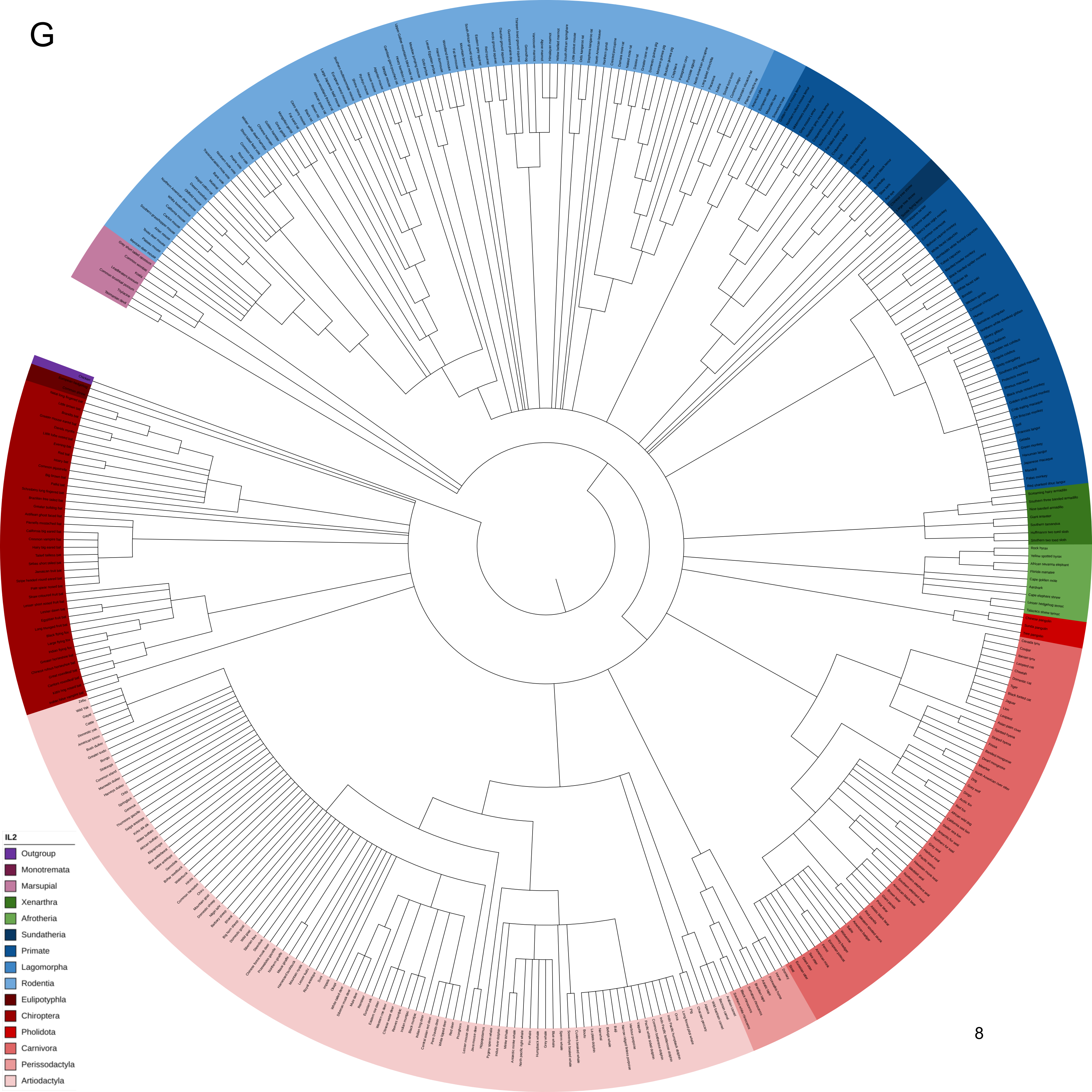
IL2
Outgroup
Monotremata
Marsupial
Xenarthra
Afrotheria
Sundatheria
Primate
Lagomorpha
Rodentia
Eulipotyphla
Chiroptera
Pholidota
Carnivora
Perissodactyla
Artiodactyla

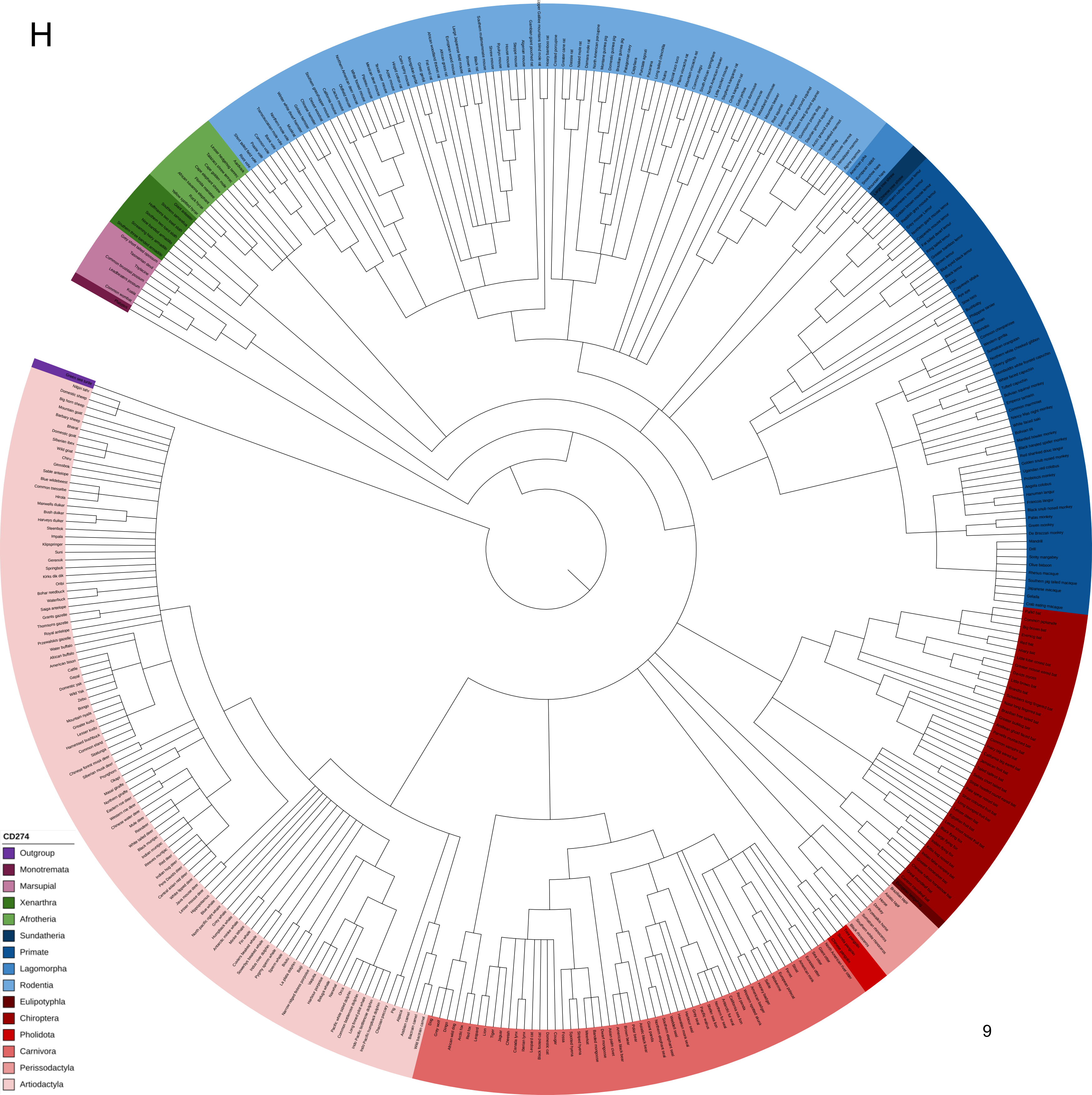
H
CD274
Outgroup
Monotremata
Marsupial
Xenarthra
Afrotheria
Sundatheria
Primate
Lagomorpha
Rodentia
Eulipotyphla
Chiroptera
Pholidota
Carnivora
Perissodactyla
Artiodactyla

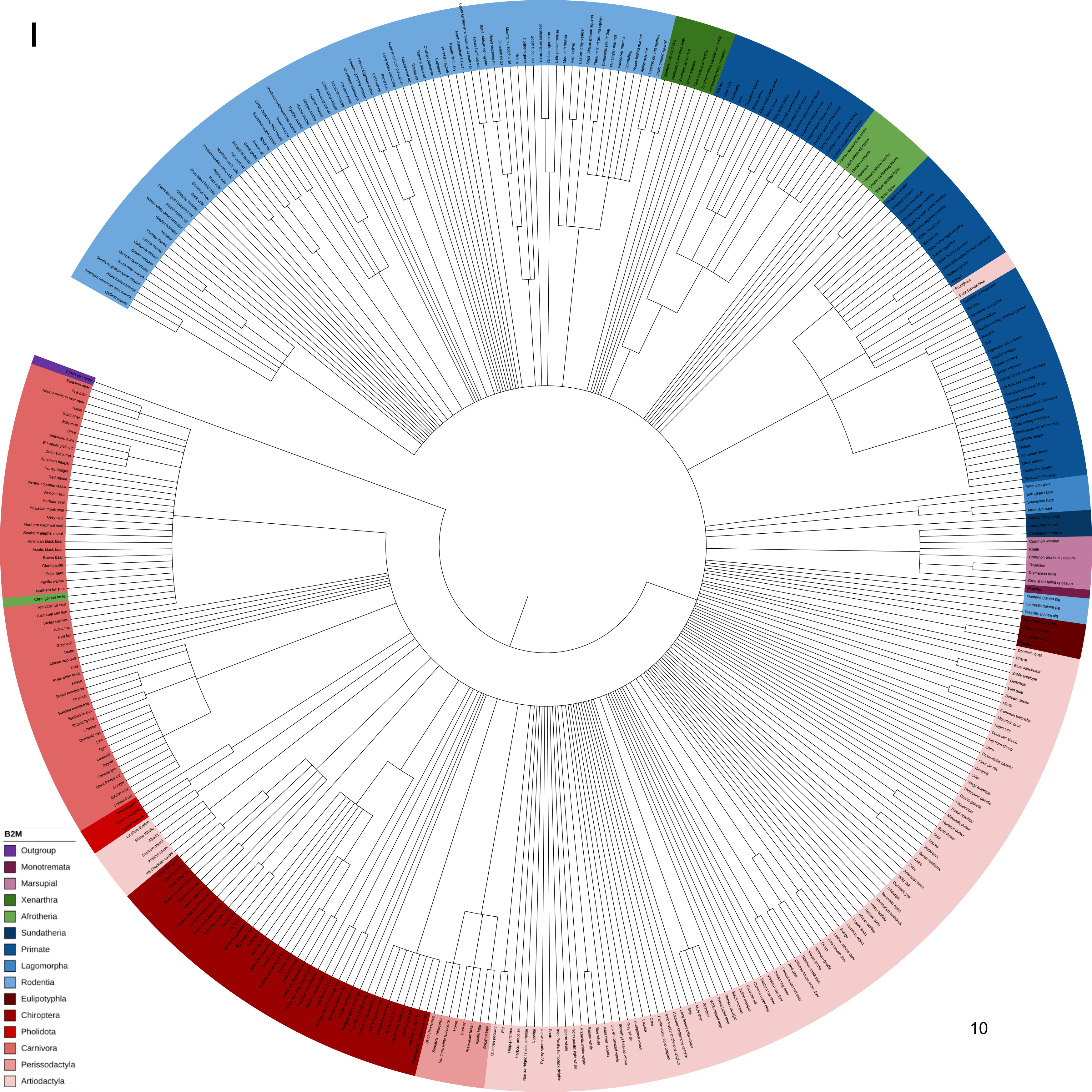
I
B2M
Outgroup
Monotremata
Marsupial
Xenarthra
Afrotheria
Sundatheria
Primate
Lagomorpha
Rodentia
Eulipotyphla
Chiroptera
Pholidota
Carnivora
Perissodactyla
Artiodactyla

Supplementary figure 1. Phylogenetic reconstructions of Mammalian cancer associated genes

Phylogenetic trees of mammalian cancer associated genes were constructed in MEGA-X[1]. Phylogenetic trees were modelled using Kimura 2-parameter model[2], and maximum likelihood analyses were run with 500 bootstrap replications and a bootstrap cut off value of 70 on the final tree. Nearest neighbour joining Tree inference was used. The phylogenetic trees were visualised using iTol V5[3]. The genes are presented as; **A.** GRB2, **B.** FGL2, **C.** CDC42, **D.** LITAF, **E.** Casp8, **F.** BRCA2,
G. IL2, **H.** CD274, **I.** B2M

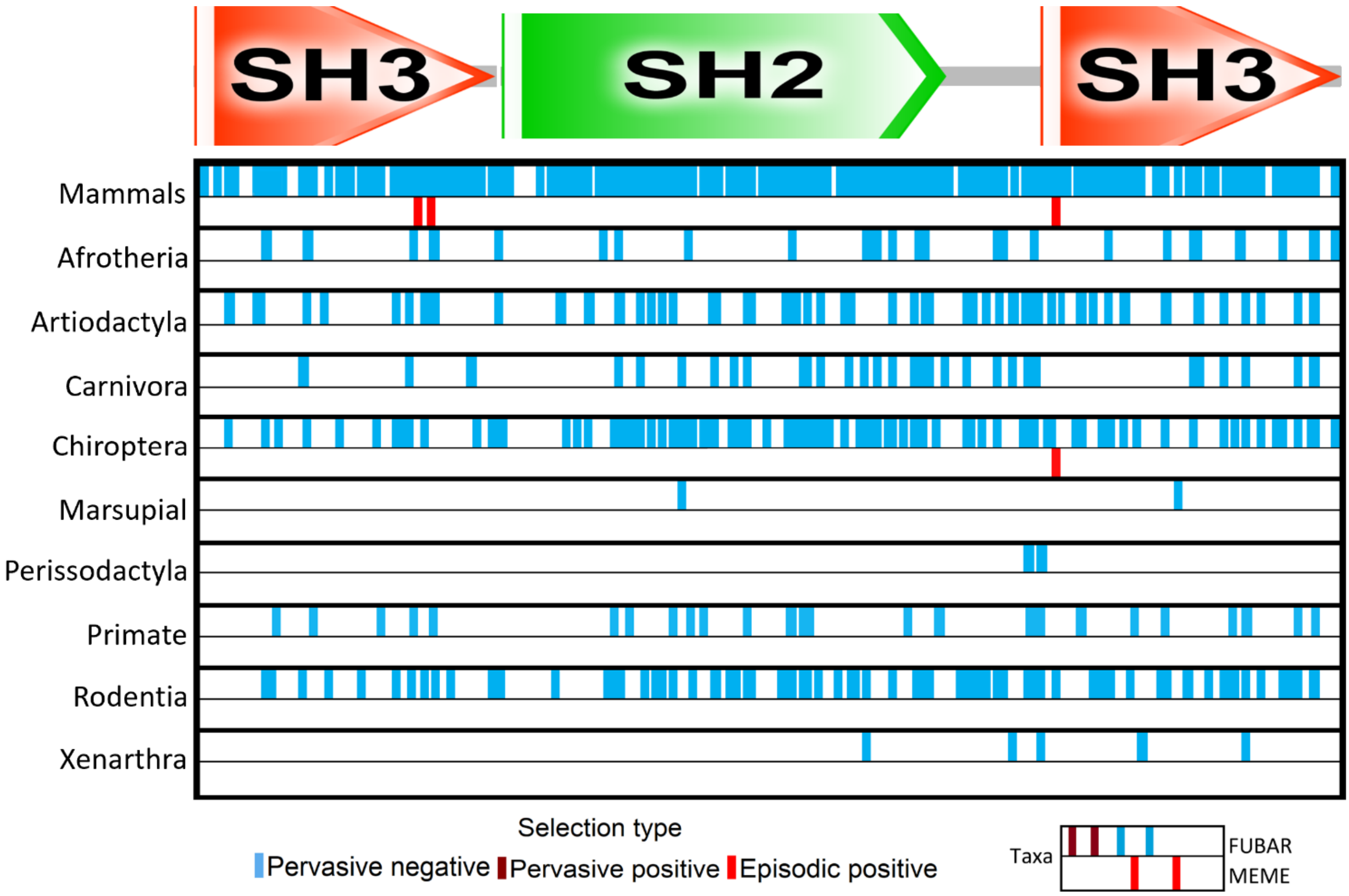

Supplementary figure 2. *GRB2 signatures of selection*

Detection of pervasive selection using Fast, Unconstrained Bayesian AppRoximation (FUBAR) and episodic positive selection using the Mixed Effects Model of Evolution (MEME) conducted on GRB2. The tests were run across different Mammalian taxa, ranging from class to order. Highlights where on the gene sequence the selection has been detected. Blue indicates negative selection and red positive selection. Domain image was sourced from the Simple Modular Architecture Research Tool and altered to represent relevant information for the gene[4].

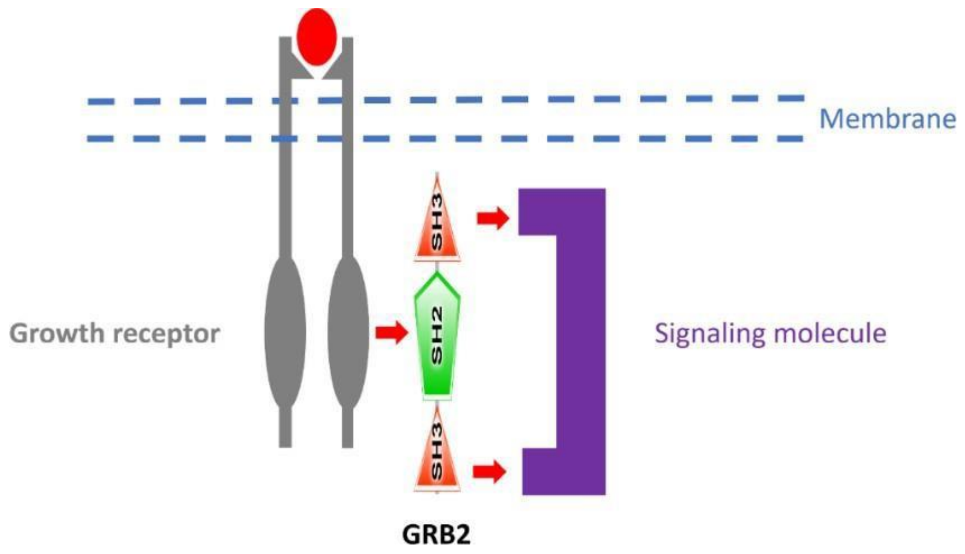

Supplementary figure 3. GRB2 functional domains

GRB2 is an adapter protein involved in various signaling pathways. The SH2 domain binds to phosphorylated tyrosine-containing peptides, which are often on transmembrane signaling molecules. The SH3 domains bind to proline rich regions. The interactions between these domains and other proteins activate proteins such as Ras[5]. Domain image was sourced from the Simple Modular Architecture Research Tool and altered to represent relevant information for the gene[4].

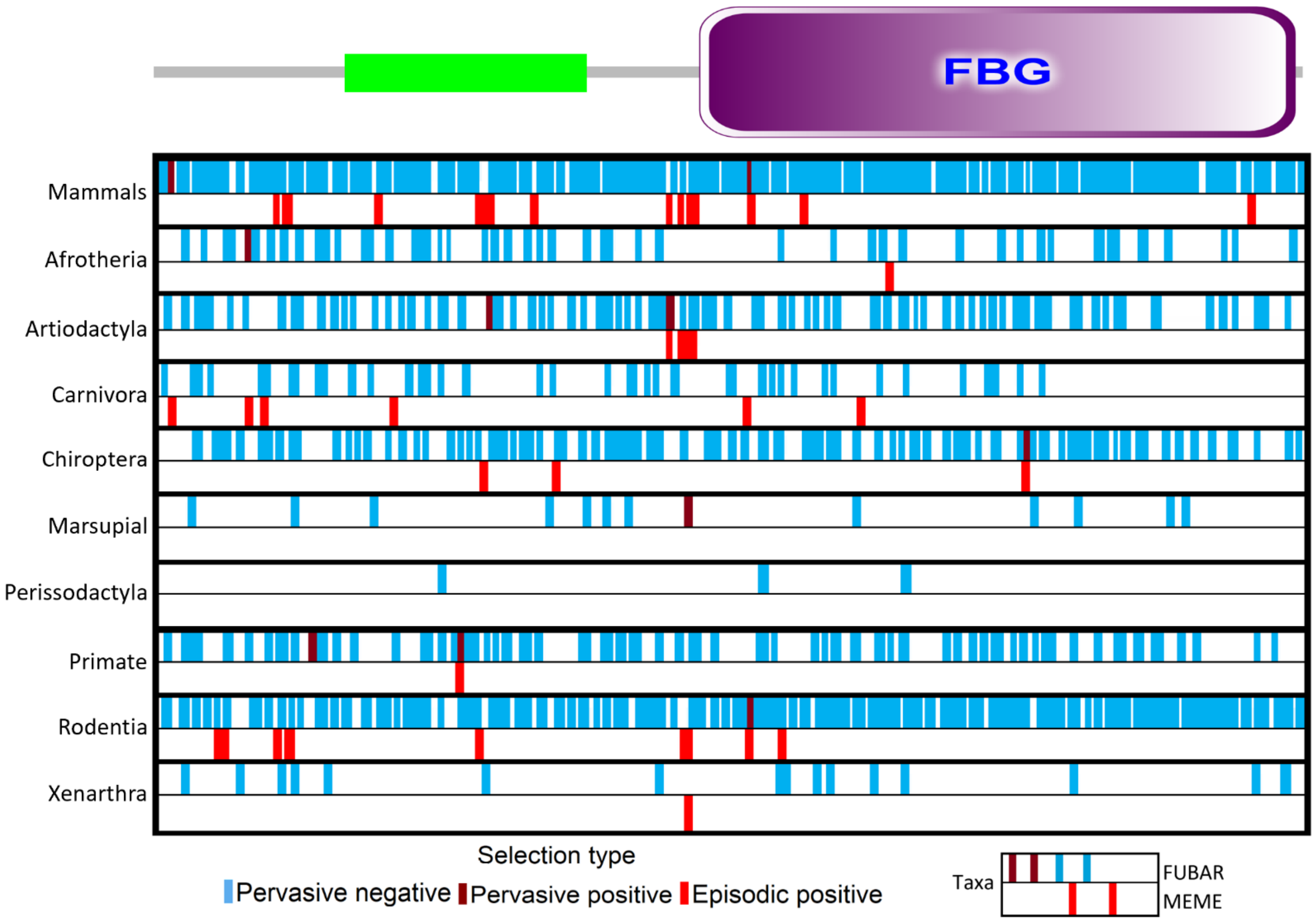

Supplementary figure 4. FGL2 Signatures of selection

Detection of pervasive selection using Fast, Unconstrained Bayesian AppRoximation (FUBAR) and episodic positive selection using the Mixed Effects Model of Evolution (MEME) was conducted on FGL2. The tests were run across different Mammalian taxa, ranging from class to order. Amino acid sites are highlighted where on the gene sequence, the selection has been detected. Blue indicates negative selection and red positive selection. Domain image was sourced from the Simple Modular Architecture Research Tool and altered to represent relevant information for the gene[4].

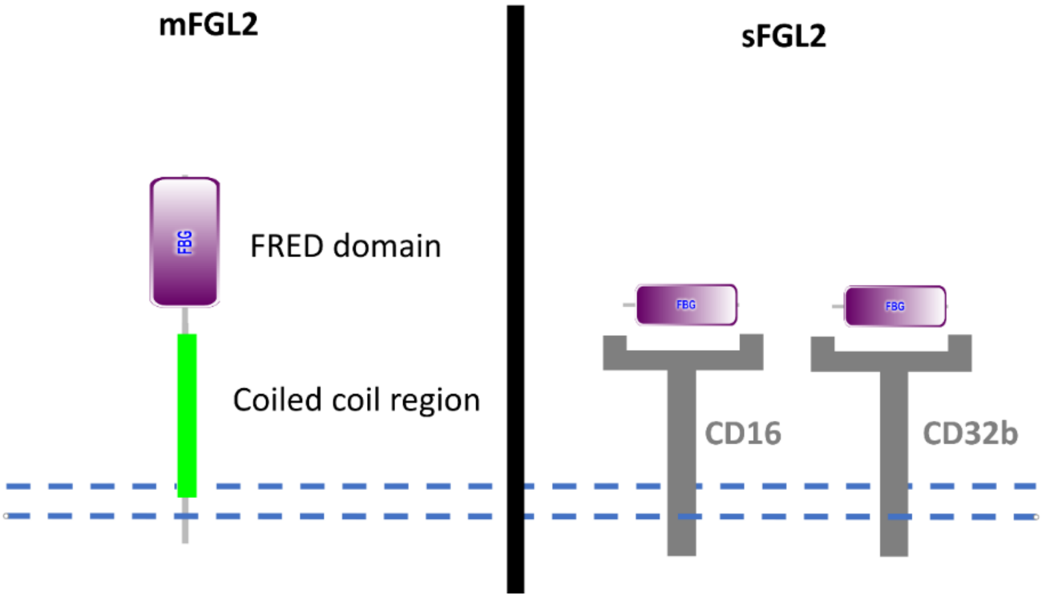

Supplementary figure 5. FGL2 functional domains

FGL2 has a membrane bound (mFGL2) and soluble (sFLG2) form that each have different functions. mFGL2 can cleave prothrombin to thrombin to promote clotting. The catalytic activity occurs in the coiled coil domain, though the structure of the FRED domain is believed to be important in this process as well, due to the loss of catalytic activity when the domain is mutated. sFGL2 acts as an immunomodulatory molecule and regulates the activity of B-cells monocytes and other immune cells by binding to the FCgamma receptors CD16 and CD32b. The soluble form consists of just the FRED domain and a section of the sequence between it and the coiled coil domain[6]. Domain image was sourced from the Simple Modular Architecture Research Tool and altered to represent relevant information for the gene[4].

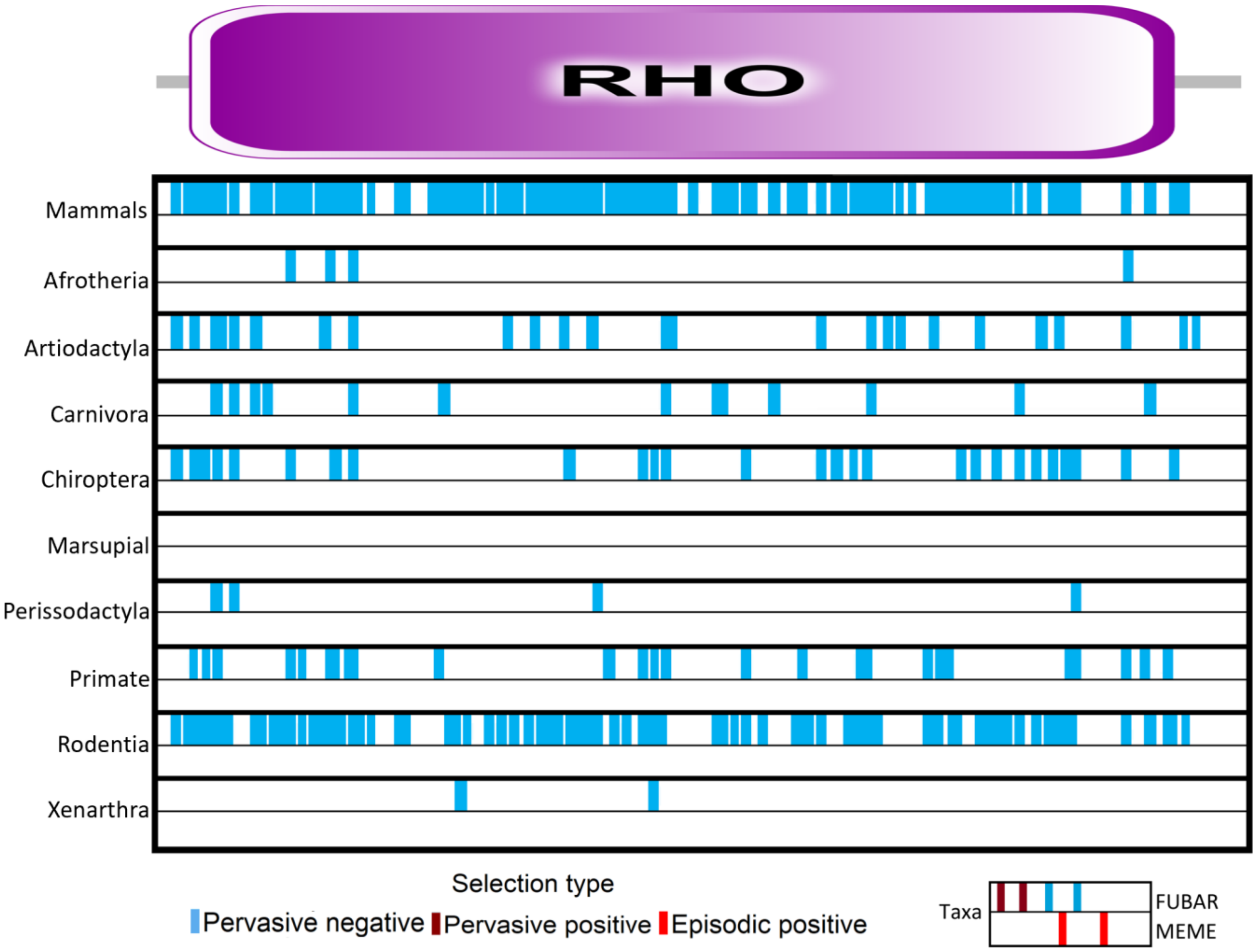

Supplementary figure 6. CDC42 Signatures of selection

Detection of pervasive selection using Fast, Unconstrained Bayesian AppRoximation (FUBAR) and episodic positive selection using the Mixed Effects Model of Evolution (MEME) was conducted on CDC42. The tests were run across different Mammalian taxa, ranging from class to order. Amino acid sites are highlighted where on the gene sequence, the selection has been detected. Blue indicates negative selection and red positive selection. Domain image was sourced from the Simple Modular Architecture Research Tool and altered to represent relevant information for the gene[4].

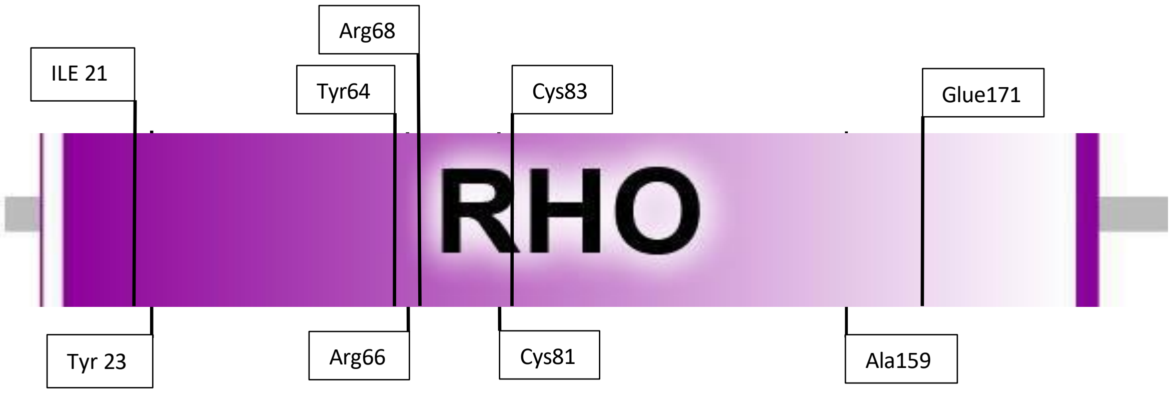

Supplementary figure 7. CDC42 Functional sites

CDC42 is a signaling molecule that is a part of the Rho GTPase family of proteins. It is involved in pathways regulating cell movement, structure, and proliferation[7]. Highlighted above are mutations known to cause disease[8,9]. Domain image was sourced from the Simple Modular Architecture Research Tool and altered to represent relevant information for the gene[4].

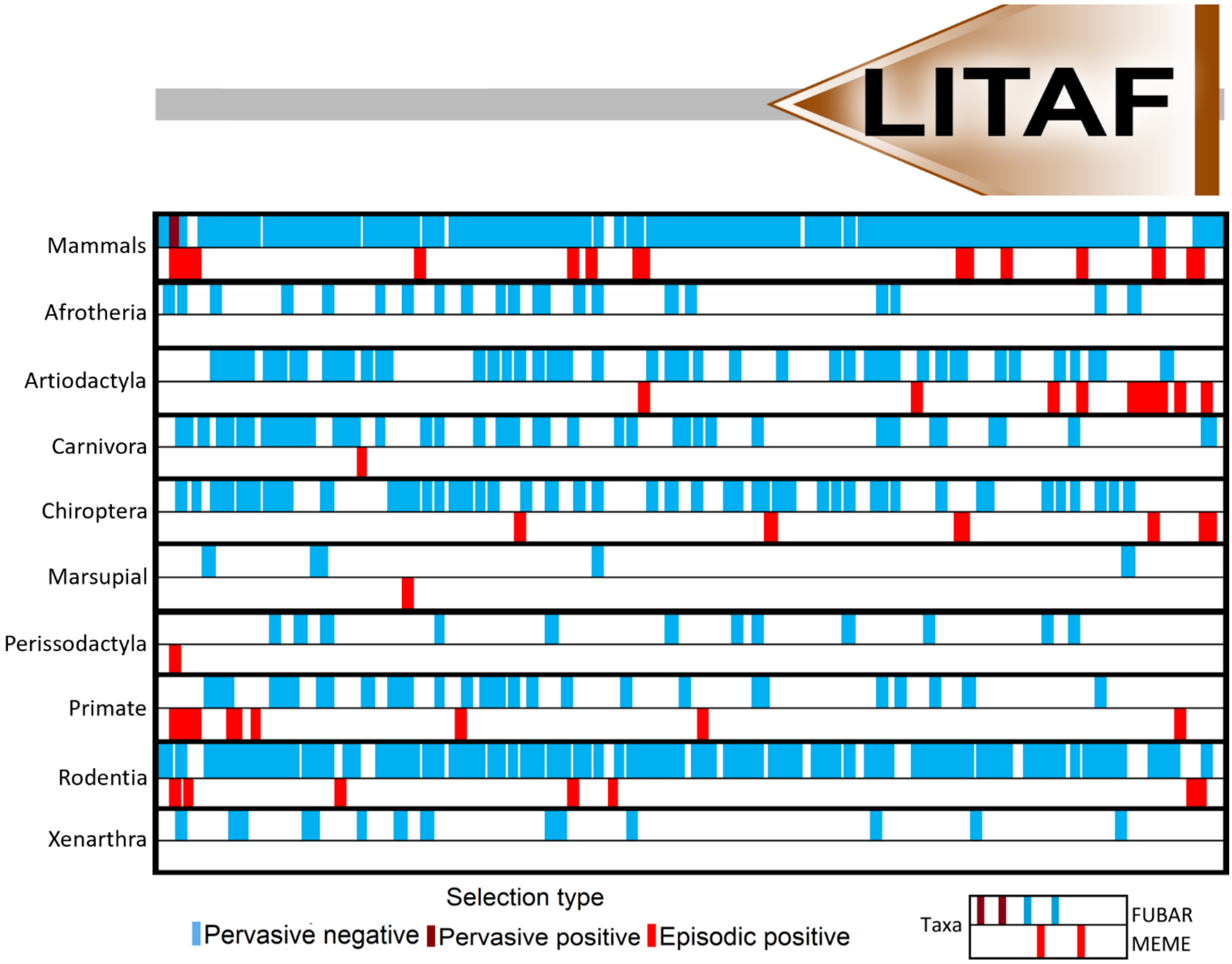

Supplementary figure 8. LITAF Signatures of selection

Detection of pervasive selection using Fast, Unconstrained Bayesian AppRoximation (FUBAR) and episodic positive selection using the Mixed Effects Model of Evolution (MEME) was conducted on LITAF. The tests were run across different Mammalian taxa, ranging from class to order. Amino acid sites are highlighted where on the gene sequence, the selection has been detected. Blue indicates negative selection and red positive selection. Domain image was sourced from the Simple Modular Architecture Research Tool and altered to represent relevant information for the gene[4].

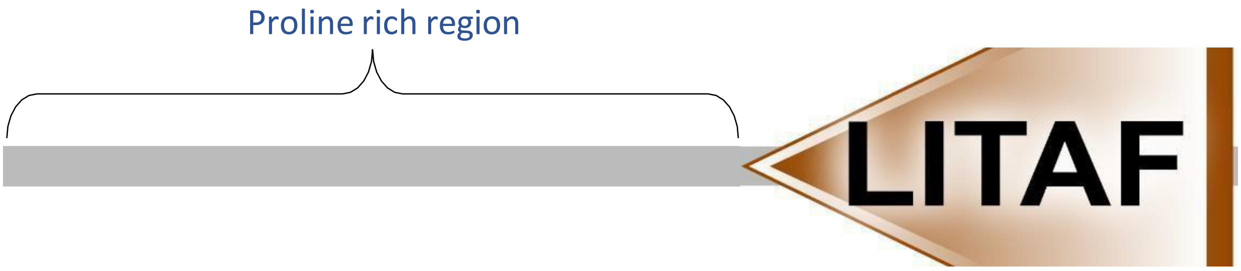

Supplementary figure 9. LITAF functional domains

LITAF domain is believed to anchor the protein in membranes and the Proline-rich region to be responsible for protein-protein interactions. Mutations causing Charcot Marie Tooth disease in humans occur in the LITAF domain[10]. Domain image was sourced from the Simple Modular Architecture Research Tool and altered to represent relevant information for the gene[4].

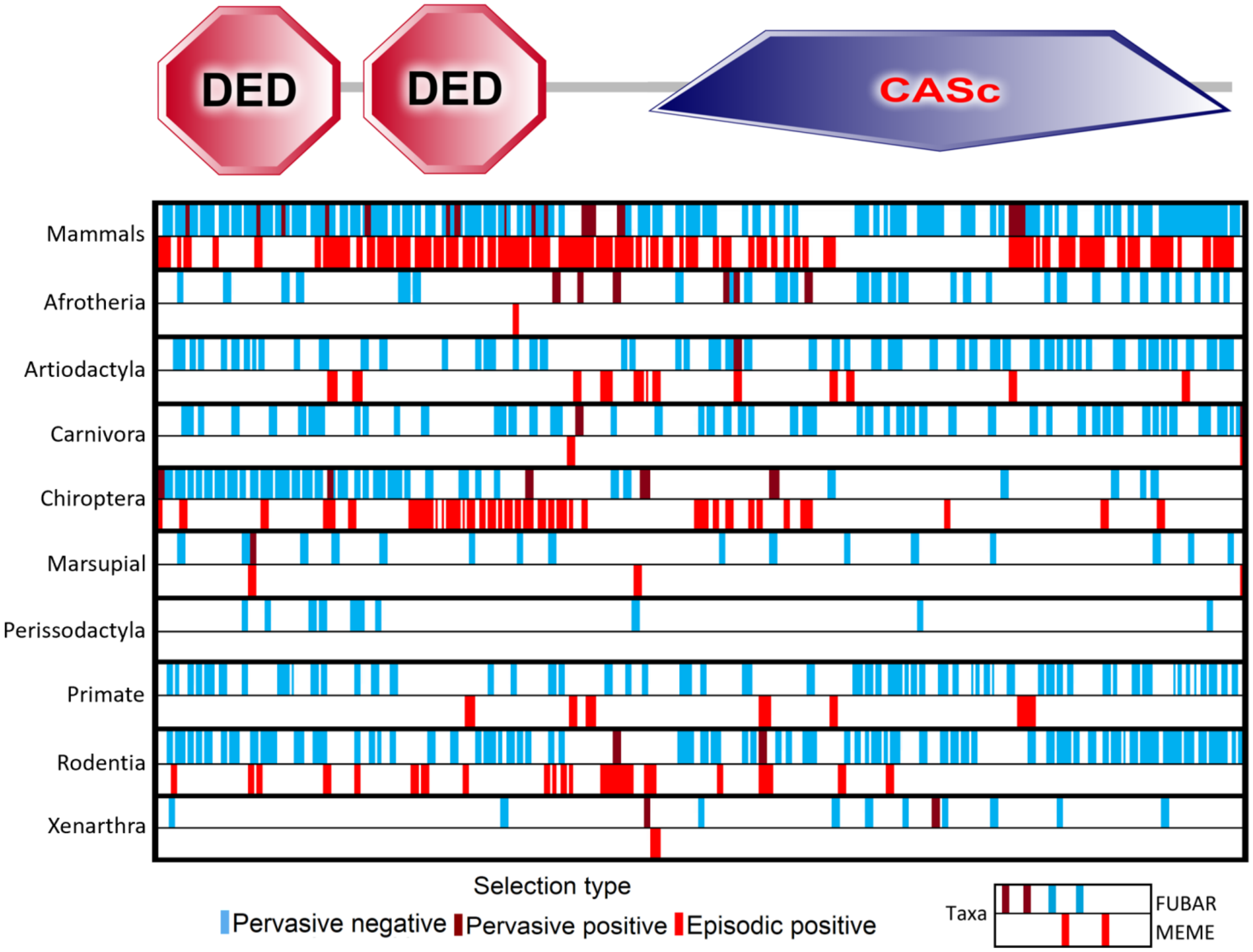

Supplementary figure 10. Casp8 Signatures of selection

Detection of pervasive selection using Fast, Unconstrained Bayesian AppRoximation (FUBAR) and episodic positive selection using the Mixed Effects Model of Evolution (MEME) was conducted on Casp8. The tests were run across different Mammalian taxa, ranging from class to order. Amino acid sites are highlighted where on the gene sequence, the selection has been detected. Blue indicates negative selection and red positive selection. Domain image was sourced from the Simple Modular Architecture Research Tool and altered to represent relevant information for the gene[4].

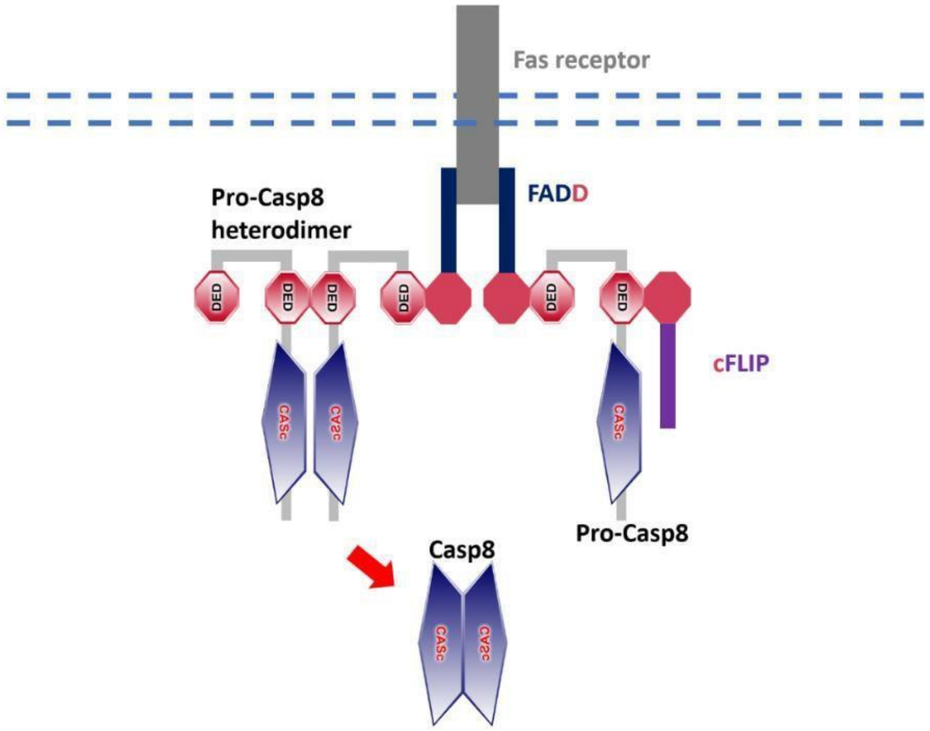

Supplementary figure 11. Casp8 functional domains

The two DED domains are responsible for Casp8 recruitment. To activate Casp8, DED1 will bind to the DED domain of the FADD protein after being recruited by the Fas receptor, and DED2 to another Casp8 protein. The Casc domains are then cleaved from the rest of the proteins and will go on to activate apoptotic pathways downstream. cFLIP can inhibit the activation of Casp8 by binding to DED2 with its DED domain[11]. Domain image was sourced from the Simple Modular Architecture Research Tool and altered to represent relevant information for the gene[4].

Figure was adapted from: Lavrik and Krammer 2009[11] and altered to represent relevant information for the gene.

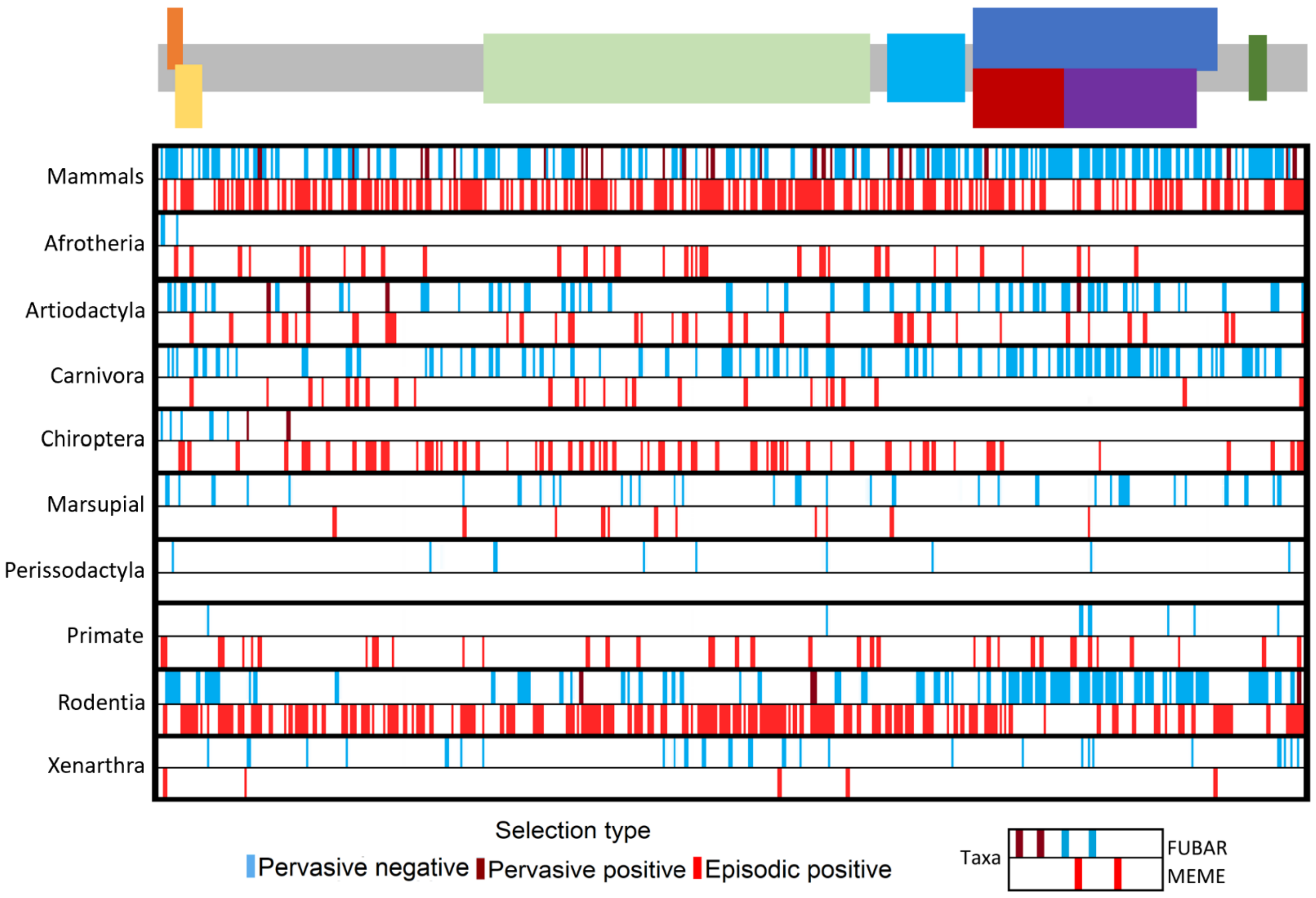

Supplementary figure 12. BRCA2 Signatures of selection

Detection of pervasive selection using Fast, Unconstrained Bayesian AppRoximation (FUBAR) and episodic positive selection using the Mixed Effects Model of Evolution (MEME) was conducted on BRCA2. The tests were run across different Mammalian taxa, ranging from class to order. Amino acid sites are highlighted where on the gene sequence, the selection has been detected. Blue indicates negative selection and red positive selection.

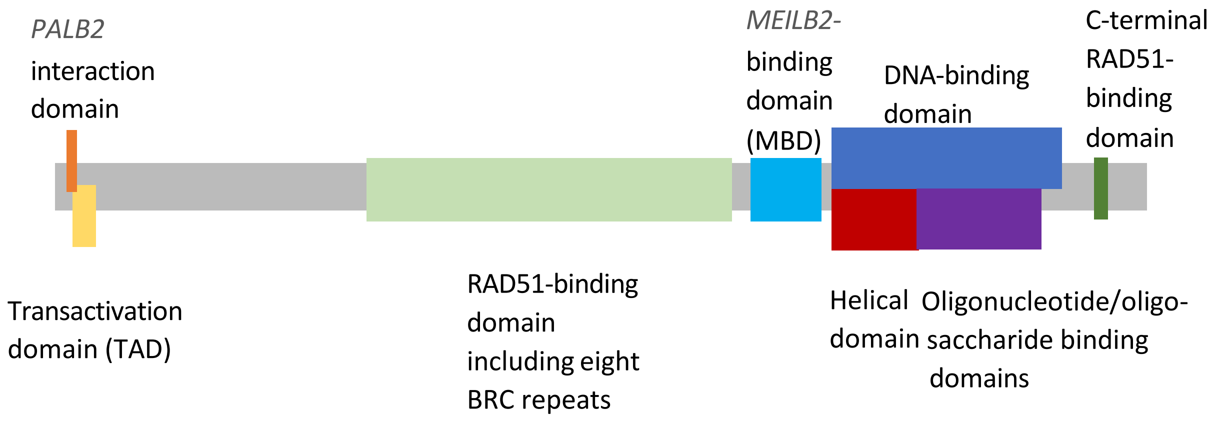

Supplementary figure 13. BRCA2 functional domains

BRCA2 is primarily involved in DNA damage repair and maintaining genomic stability. It's a large gene (3418 aa in humans) and has many domains that interact with different pathways[12,13].

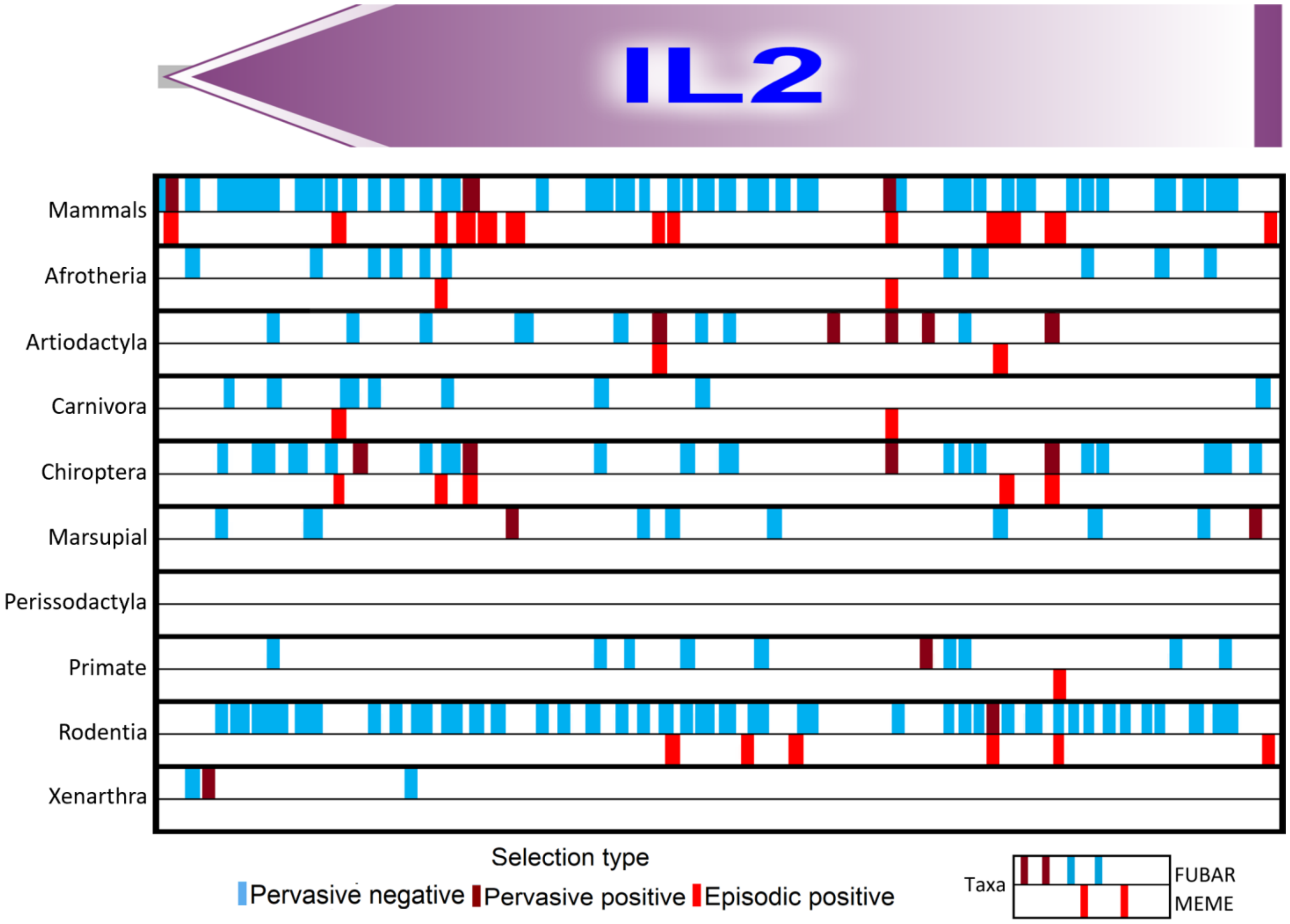

Supplementary figure 14. IL2 Signatures of selection

Detection of pervasive selection using Fast, Unconstrained Bayesian AppRoximation (FUBAR) and episodic positive selection using the Mixed Effects Model of Evolution (MEME) was conducted on IL2. The tests were run across different Mammalian taxa, ranging from class to order. Amino acid sites are highlighted where on the gene sequence, the selection has been detected. Blue indicates negative selection and red positive selection. Domain image was sourced from the Simple Modular Architecture Research Too and altered to represent relevant information for the gene[4].

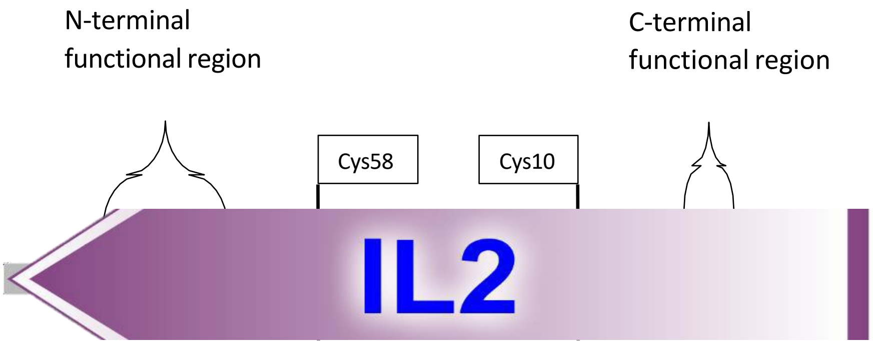

Supplementary figure 15. IL2 functional domains

Multiple sites of IL2 are important and when mutated the protein loses its capacity to function. The N and C terminal functional regions and the Cysteine residues at sites 58 and 105 are adjacent to each other when the protein is folded in post-translation modification. Together they form the region of IL2 that interreacts with the IL2 receptor[14]. Domain image was sourced from the Simple Modular Architecture Research Tool and altered to represent relevant information for the gen[4].

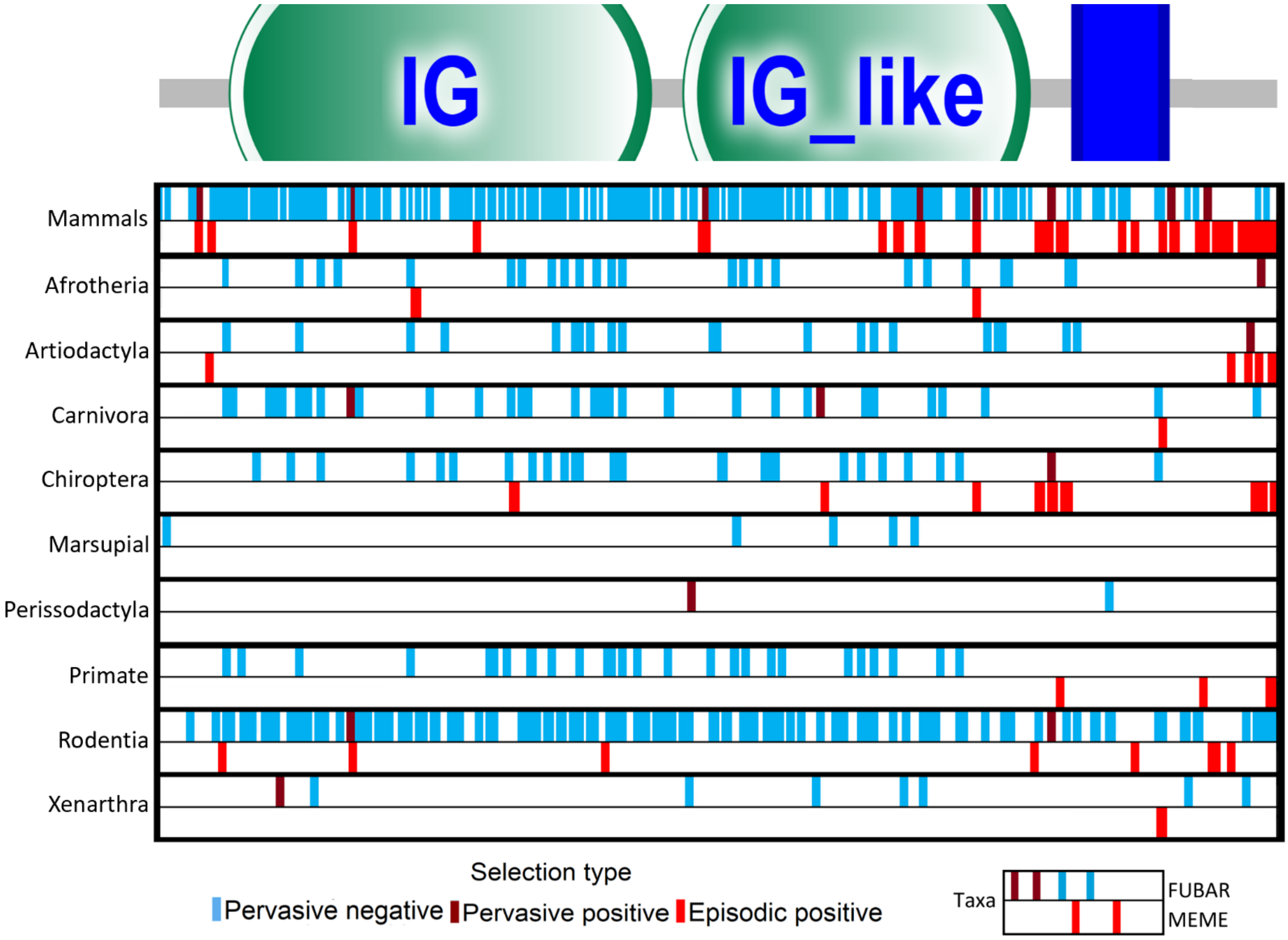

Supplementary figure 16. CD274 Signatures of selection

Detection of pervasive selection using Fast, Unconstrained Bayesian AppRoximation (FUBAR) and episodic positive selection using the Mixed Effects Model of Evolution (MEME) was conducted on CD274. The tests were run across different Mammalian taxa, ranging from class to order. Amino acid sites are highlighted where on the gene sequence, the selection has been detected. Blue indicates negative selection and red positive selection. Domain image was sourced from the Simple Modular Architecture Research Tool and altered to represent relevant information for the gene[4].

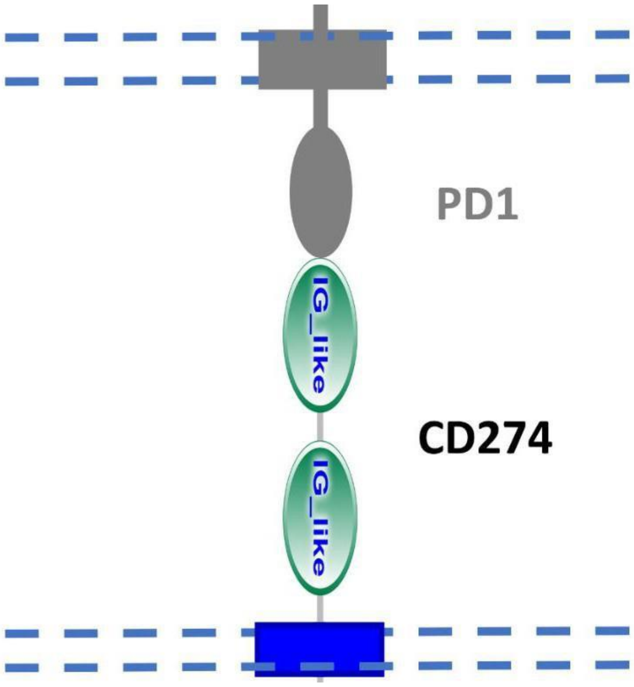

Supplementary figure 17. CD274 functional domains

CD274 is an external signalling molecule anchored to the cell by its C-terminal transmembrane region. The PD-1 receptor molecule binds to the first Ig-like domain to transduce the inhibitory signal on the cell, while the second Ig-like domain remains outside the interaction zone[15]. Domain image was sourced from the Simple Modular Architecture Research Tool and altered to represent relevant information for the gene[4].

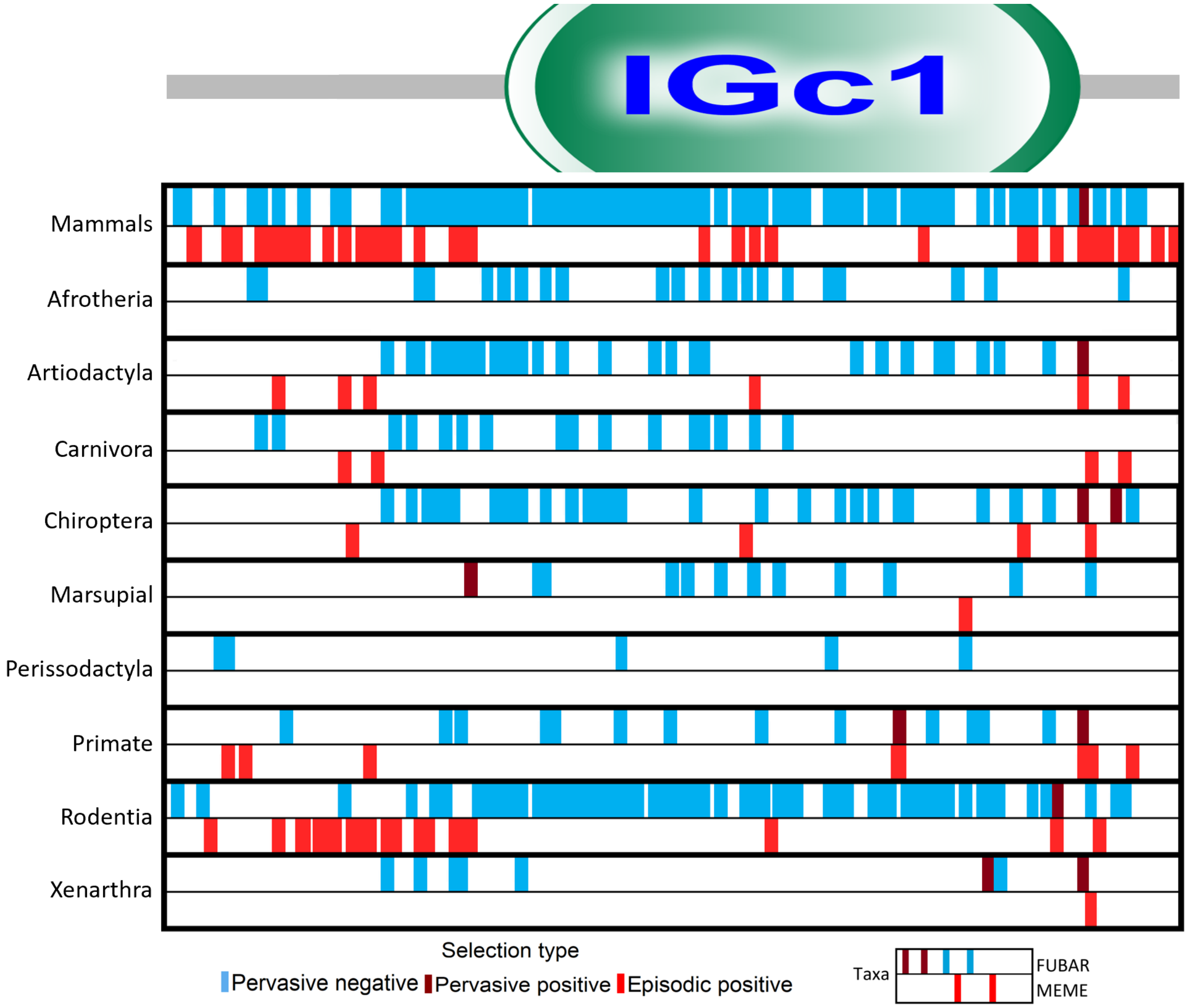

Supplementary figure 18. B2M Signatures of selection

Detection of pervasive selection using Fast, Unconstrained Bayesian AppRoximation (FUBAR) and episodic positive selection using the Mixed Effects Model of Evolution (MEME) was conducted on B2M. The tests were run across different Mammalian taxa, ranging from class to order. Amino acid sites are highlighted where on the gene sequence, the selection has been detected. Blue indicates negative selection and red positive selection. Domain image was sourced from the Simple Modular Architecture Research Tool and altered to represent relevant information for the gene[4].

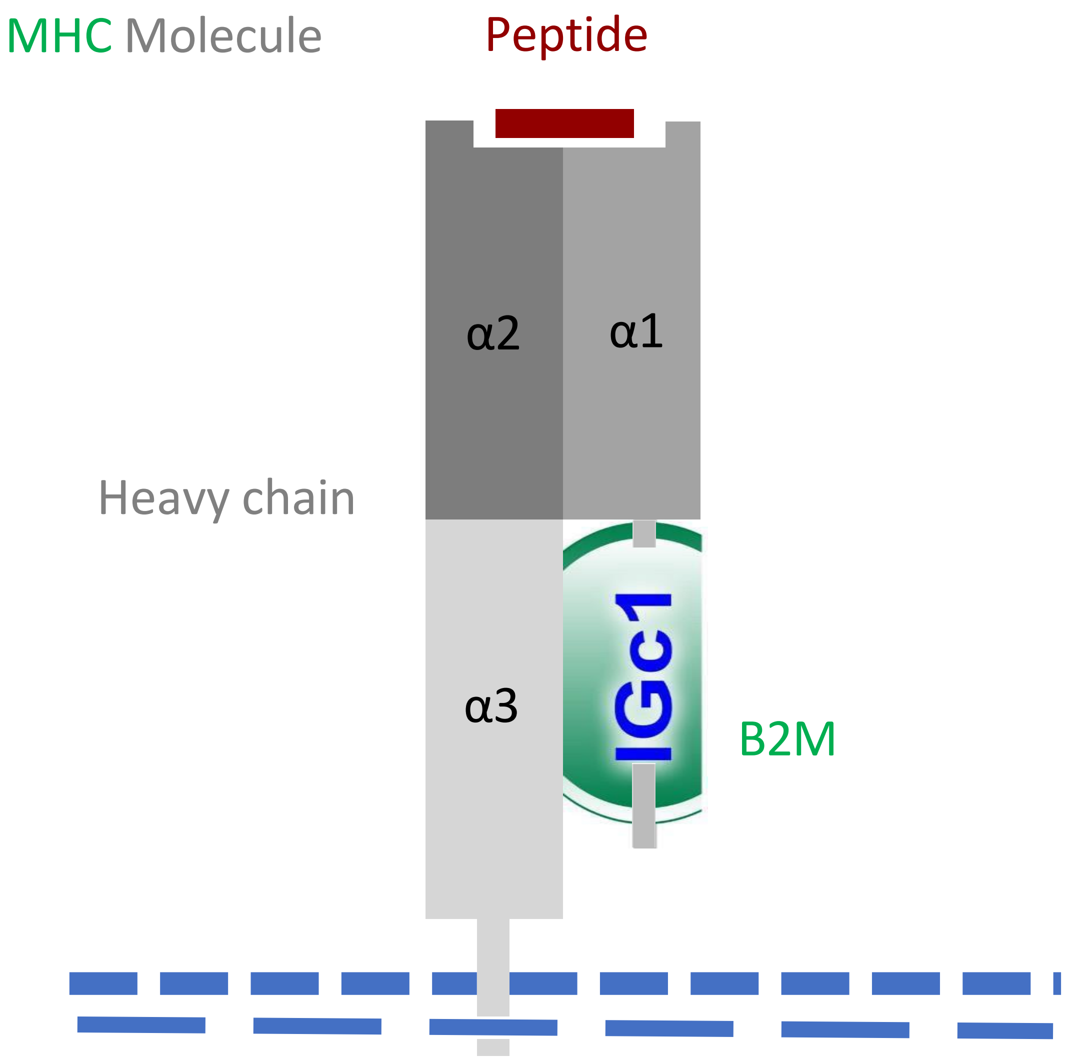

Supplementary figure 19. B2M functional domains

B2M is a subunit of the MHC class 1 molecule. During the process of antigen presentation, it is attached to the heavy chain, which consists of the α1,2 and 3, domains. Before forming the MHC molecule, B2M folds into a structure consisting of two β-sheets and a di-sulfide bond holding them together[16]. Domain image was sourced from the Simple Modular Architecture Research Tool and altered to represent relevant information for the gene[4].

Supplementary table 1. Logistic regression model: effect of sample size on the detection of amino acid sites under selection

The table shows the output of the logistic regression model on the effect of sample size on the number of sites under either negative or positive selection detected using the 'Fast, Unconstrained Bayesian AppRoximation for Inferring Selection' and 'Mixed effects model of evolution' statistical programs. The model was run across the nine cancer associated genes; GRB2, FGL2, CDC42, LITAF, Casp8, BRCA2, IL2, CD274 and B2M and it can be concluded that for these genes the sample size does not affect the number of sites detected by the statistical programs.

Gene	Coefficients	Estimate	Standard deviation	Z-test	Pr(>|z|)
GRB2	(Intercept)	-2.609711	0.121061	-21.56	<2e-16
	Sample size	0.018891	0.001811	10.43	<2e-16
FGL2	(Intercept)	-2.663878	0.084601	-31.49	<2e-16
	Sample size	0.022537	0.001219	18.49	<2e-16
CDC42	(Intercept)	-3.359308	0.172620	-19.461	<2e-16
	Sample size	0.021281	0.002414	8.816	<2e-16
LITAF	(Intercept)	-2.282920	0.120975	-18.87	<2e-16
	Sample size	0.025893	0.001892	13.68	<2e-16
Casp8	(Intercept)	-2.653396	0.083954	-31.61	<2e-16
	Sample size	0.017082	0.001262	13.54	<2e-16
BRCA2	(Intercept)	-4.3557040	0.0653853	-66.62	<2e-16
	Sample size	0.0204755	0.0008994	22.77	<2e-16
IL2	(Intercept)	-2.898918	0.165728	-17.492	<2e-16
	Sample size	0.016785	0.002447	6.859	6.92e-12
CD274	(Intercept)	-3.060873	0.124615	-24.56	<2e-16
	Sample size	0.020101	0.001789	11.23	<2e-16
B2M	(Intercept)	-2.397709	0.141719	-16.92	<2e-16
	Sample size	0.023243	0.002278	10.20	<2e-16

Supplementary table 2. Chi squared test; sample size on sites number of sites under selection.

Chi squared test was conducted to assess the effect of sample size on the number of sites under either negative or positive selection detected by the 'Fast, Unconstrained Bayesian AppRoximation for Inferring Selection' and 'Mixed effects model of evolution' statistical programs. The model was run across the nine cancer associated genes; GRB2, FGL2, CDC42, LITAF, Casp8, BRCA2, IL2, CD274 and B2M and it can be concluded that for these genes the sample size does not affect the number of sites detected by the statistical programs. This model accounted for the variation in amino acids numbers across each gene.

Gene	Chi squared	Degrees of freedom	p-value
GRB2	264.92	8	< 2.2e-16
FGL2	656.47	8	< 2.2e-16
CDC42	136.2	8	< 2.2e-16
LITAF	316.83	8	< 2.2e-16
Casp8	350.44	8	< 2.2e-16
BRCA2	1391.2	8	< 2.2e-16
IL2	160.29	8	< 2.2e-16
CD274	376.99	8	< 2.2e-16
B2M	215.86	8	< 2.2e-16

Supplementary table 3. Chi squared posthoc effect of sample size on sites number of sites under selection by sub- group.

Chi squared posthoc test was conducted to test the effect of sample size on the number of sites under either negative or positive selection when detected by the 'Fast, Unconstrained Bayesian AppRoximation for Inferring Selection' and 'Mixed effects model of evolution' statistical programs. The model was run across the nine cancer associated genes; GRB2, FGL2, CDC42, LITAF, Casp8, BRCA2, IL2, CD274 and B2M and it can be concluded that for these genes the sample size does not affect the number of sites detected by the statistical programs. This model took into account the differences in amino acids numbers for each gene.

Group	Value	GRB2	FGL2	CDC42	LITAF	Casp8	BRCA2	IL2	CD274	B2M
Afrotheria	Residuals	-1.584	-3.241	-0.553	-3.934	-3.323	-7.374	-1.170	-1.236	-3.644
	p-value	1.000	0.021	1.000	0.001	0.016	0.000	1.000	1.000	0.004
Artiodactyla	Residuals	-3.961	3.381	3.638	3.978	2.554	-0.679	-0.358	-1.432	2.159
	p-value	0.001	0.012	0.004	0.001	0.191	1.000	1.000	1.000	0.554
Carnivora	Residuals	-0.198	3.950	-1.600	0.363	0.175	4.172	-1.711	-0.065	-1.169
	p-value	1.000	0.001	1.000	1.000	1.000	>0.001	1.000	1.000	1.000
Chiroptera	Residuals	10.498	6.002	-1.076	3.217	8.710	0.291	3.702	1.497	2.397
	p-value	0.000	0.000	1.000	0.023	0.000	1.000	0.003	1.000	0.297
Marsupial	Residuals	-6.338	-8.698	-3.172	-6.834	-6.542	-6.888	1.395	-5.532	-2.833
	p-value	0.000	0.000	0.027	0.000	0.000	0.000	1.000	0.000	0.0827
Perissodactyla	Residuals	-6.338	-9.999	-3.696	-5.534	-7.801	-11.061	-4.603	-6.118	-4.736
	p-value	0.000	0.000	0.003	0.000	0.000	0.000	0.000	0.000	0.000
Primate	Residuals	-1.584	1.408	0.232	-0.397	2.415	-5.142	-1.711	-0.455	-0.693
	p-value	1.000	1.000	1.000	1.000	0.283	>0.001	1.000	1.000	1.000

Rodentia	Residuals	7.328	20.723	9.925	14.443	11.512	35.0265	11.011	18.095	13.098
	p-value	0.000	0.000	0.000	0.000	0.000	0.000	0.000	0.000	0.000
Xenarthra	Residuals	-5.744	-8.360	-3.696	-5.343	-7.661	-8.344<br>0.000	-3.877	-4.751	-3.785
	p-value	0.000	0.000	0.003	0.000	0.000	0.000	0.001	0.000	0.002

References

1 Kumar, S., Stecher, G., Li, M., Knyaz, C. & Tamura, K. MEGA X: molecular evolutionary genetics analysis across computing platforms. *Molecular biology and evolution* **35**, 1547 (2018).

2 Kimura, M. A simple method for estimating evolutionary rates of base substitutions through comparative studies of nucleotide sequences. *Journal of molecular evolution* **16**, 111-120 (1980).

3 Letunic, I. & Bork, P. Interactive Tree Of Life (iTOL) v5: an online tool for phylogenetic tree display and annotation. *Nucleic acids research* **49**, W293-W296 (2021).

4 Schultz, J., Milpetz, F., Bork, P. & Ponting, C. P. SMART, a simple modular architecture research tool: identification of signaling domains. *Proceedings of the National Academy of Sciences* **95**, 5857-5864 (1998).

5 Chardin, P., Cussac, D., Maignan, S. & Ducruix, A. The GRB2 adaptor. *FEBS letters* **369**, 47-51 (1995).

6 Liu, X.-g., Liu, Y. & Chen, F. Soluble fibrinogen like protein 2 (sFGL2), the novel effector molecule for immunoregulation. *Oncotarget* **8**, 3711 (2017).

7 Melendez, J., Grogg, M. & Zheng, Y. Signaling role of Cdc42 in regulating mammalian physiology. *Journal of Biological Chemistry* **286**, 2375-2381 (2011).

8 Martinelli, S. *et al.* Functional dysregulation of CDC42 causes diverse developmental phenotypes. *The American Journal of Human Genetics* **102**, 309-320 (2018).

9 Szczawinska-Poplonyk, A., Ploski, R., Bernatowska, E. & Pac, M. A novel CDC42 mutation in an 11-year old child manifesting as syndromic immunodeficiency, autoinflammation, hemophagocytic lymphohistiocytosis, and malignancy: a case report. *Frontiers in Immunology* **11**, 516597 (2020).

10 Ho, A. K. *et al.* The topology, structure and PE interaction of LITAF underpin a Charcot-Marie-Tooth disease type 1C. *BMC biology* **14**, 1-21 (2016).

11 Lavrik, I. N. & Krammer, P. H. Life and death decisions in the cd95 system: main pro-and anti-apoptotic modulators. *Acta naturae* **1**, 80 (2009).

12 Fradet-Turcotte, A., Sitz, J., Grapton, D. & Orthwein, A. BRCA2 functions: from DNA repair to replication fork stabilization. *Endocr Relat Cancer* **23**, T1-T17 (2016).

13 Ikegami, M. *et al.* High-throughput functional evaluation of BRCA2 variants of unknown significance. *Nature communications* **11**, 2573 (2020).

14 Ju, G. *et al.* Structure-function analysis of human interleukin-2. Identification of amino acid residues required for biological activity. *Journal of Biological Chemistry* **262**, 5723-5731 (1987).

15 Zak, K. M. *et al.* Structural biology of the immune checkpoint receptor PD-1 and its ligands PD-L1/PD-L2. *Structure* **25**, 1163-1174 (2017).

16 Ami, D. *et al.* Structure, stability, and aggregation of β-2 microglobulin mutants: insights from a Fourier transform infrared study in solution and in the crystalline state. *Biophysical journal* **102**, 1676-1684 (2012).
